# Protein orientation in time-dependent electric fields: orientation before destruction

**DOI:** 10.1016/j.bpj.2021.07.017

**Published:** 2021-07-23

**Authors:** Anna Sinelnikova, Thomas Mandl, Harald Agelii, Oscar Grånäs, Erik G. Marklund, Carl Caleman, Emiliano De Santis

**Affiliations:** 1 Department of Physics and Astronomy, Uppsala University, Uppsala, Sweden; 2 University of Applied Sciences Technikum Wien, Wien, Austria; 3 Department of Chemistry BMC, Uppsala University, Uppsala, Sweden; 4 Center for Free-Electron Laser Science, DESY, Hamburg, Germany

## Abstract

Proteins often have nonzero electric dipole moments, making them interact with external electric fields and offering a means for controlling their orientation. One application that is known to benefit from orientation control is single-particle imaging with x-ray free-electron lasers, in which diffraction is recorded from proteins in the gas phase to determine their structures. To this point, theoretical investigations into this phenomenon have assumed that the field experienced by the proteins is constant or a perfect step function, whereas any real-world pulse will be smooth. Here, we explore the possibility of orienting gas-phase proteins using time-dependent electric fields. We performed ab initio simulations to estimate the field strength required to break protein bonds, with 45 V/nm as a breaking point value. We then simulated ubiquitin in time-dependent electric fields using classical molecular dynamics. The minimal field strength required for orientation within 10 ns was on the order of 0.5 V/nm. Although high fields can be destructive for the structure, the structures in our simulations were preserved until orientation was achieved regardless of field strength, a principle we denote “orientation before destruction.”

## Significance

New means for controlling molecules enable new science and applications. Recent investigations show that the orientation of proteins can be controlled with electric fields in vacuum, but the fields have been assumed to be turned on instantaneously, which is a poor description of the field experienced by a protein in a laboratory setting. Here, we instead explore the possibility of orienting proteins with time-dependent electric fields, using quantum-mechanics calculations to test the integrity of covalent bonds and classical simulations to monitor orientation and preservation of the protein structures. Our results advance our understanding of the process of dipole orientation and provide a more realistic picture upon which to base the design of an experimental apparatus to take this phenomenon from theory to practice.

## Introduction

New means for manipulating macromolecules can be of great utility for both applications and basic research in the physical and life sciences. Using classical molecular dynamics (MD) simulations, we recently demonstrated the feasibility of controlling the orientation of gas-phase proteins using a strong electric field (EF) (1). Proteins often carry an electric dipole moment (2), which interacts with the EF to generate a torque on the protein. Using EFs to manipulate proteins is not novel per se; strong EFs were used to cause domain motions in crystals of proteins in x-ray crystallography (3), and EF interactions underpin numerous separation techniques in both solution and gas phase. The field can be destructive for the protein structures, as positively and negatively charged moieties will be pulled in opposite directions, potentially leading to unfolding. Deliberate unfolding can in some cases be desired because it can inform about a protein’s structural and mechanical properties (4, 5, 6, 7), and EFs can be used to this end (8), but many applications might require native or native-like structures. If an appropriate EF strength is chosen, however, the proteins orient without significant structural loss, enabling orientation control as part of protein investigations in the gas phase. Proteins are normally evolved for aqueous solutions, and the gas-phase conditions alone can be destructive for their structures. The protein backbone is, however, believed to remain folded on short timescales (9,10), which is supported by a growing mass of evidence from simulations and experiments (9,11, 12, 13, 14, 15, 16, 17, 18, 19). Such kinetic trapping of the structures enables delivery of intact gas-phase proteins for ion mobility or mass spectrometry, or into an XFEL beam.

One application that benefits from orientation control of proteins is single-particle imaging (SPI; also “flash x-ray imaging”) (1,20), which is a technique for structure determination in which single proteins or other nanoscale particles are exposed to x-ray pulses and the resulting diffraction is used to reconstruct the three-dimensional (3D) structure of the objects under study (21,22). Unlike x-ray crystallography, which has been the dominant technique in structural biology for decades, SPI does not require crystalline samples, which enables imaging of proteins that do not crystallize at all or do not crystallize in the states of interest, e.g., because of inherent dynamics or polydispersity. To get enough diffraction from a single protein, the pulses need to be ultraintense, which quickly obliterates the protein, and the next diffraction pattern is recorded from another identical copy of the protein. The high intensity means that the pulses also need to be ultrashort so that they scatter from the unperturbed protein structure before radiation damage has time to build up, a principle that has become known as “diffraction before destruction.” Using ultrashort pulses (tens of femtoseconds) gives SPI a tremendous potential to study dynamics and kinetics in protein structures, for example, using photoexcitation to trigger a reaction just before XFEL beam exposure or fast mixing just before injection (23), in which the kinetic trapping serves to carry over the structural ensemble from solution to the gas phase. These extreme requirements are met by x-ray free-electron lasers (XFELs), such as Linac Coherent Light Source (LCLS) (24) and the European XFEL (25), which has enabled 3D imaging of the Mimi virus, revealing not only its capsid but also the genome inside, albeit at low resolution (26). Subsequent experiments have revealed the 3D structures of additional viruses (27, 28, 29) as well as carboxysomes (30).

The proteins enter the beam randomly oriented in SPI, which complicates the process of assembling the diffraction patterns into a self-consistent 3D data set—the “orientation recovery.” We have shown that prior knowledge about the orientation can support the orientation recovery, even if that knowledge is incomplete (1). Importantly, it can make the orientation-recovery algorithms converge with fewer diffraction patterns and cope better with missing data. This can make the difference between successful and failed experiments, yield better structure models, and reduce sample consumption and beam time. To this point, EF orientation has only been explored assuming static fields or step functions, but any real instrument will inevitably have a smooth pulse profile (see, e.g., Wilks et al. (31)). As such, to harness field orientation in experiments, the effects of time-dependent EFs need to be investigated. Moreover, the immediate onset of the EF might not be optimal for preserving the structure, which further motivates us to here investigate how time-varying EFs perform in orienting proteins in the gas phase and what they do to the structures. We apply a multiscale approach comprising both ab initio and classical MD simulations. Using time-dependent density functional theory in the presence of an EF, we identify the upper field strength at which covalent bonds can remain intact in a protein, defining a validity limit for classical MD. We moreover performed gas-phase classical MD simulations on ubiquitin to study the effect of time-dependent EFs on its orientation and the consequent structural evolution. Our results are key for understanding the process of dipole orientation induced by an external, time-dependent EF. As such, they can serve to guide in the design of an apparatus able to control this phenomenon to manipulate proteins in SPI and other applications.

## Materials and methods

### Ab initio MD simulations

The strong EFs applied in this study create rather extreme conditions for protein molecules, and one might ask whether EF orientation can be explored using classical models. We therefore carry out ab initio MD simulations to evaluate the impact on the integrity of covalent bonds and on the overall electron distribution. A molecule becomes polarized when subjected to external EFs as the charge in the molecule rearrange to screen the EF. Consequently, this generally changes the molecular dipole. Moreover, the rearrangements of the electronic structure result in residual forces on charged sites in the protein. We used the ab initio MD software package Siesta 4.1 (32) to estimate the forces resulting from the interaction with the EF. We followed the same procedure as published in our earlier work (8). Ab initio calculations were carried out on a small protein, Trp-cage (Protein Data Bank: 1L2Y) (33), with a total charge of +2 *e*, as is expected for Trp-cage aerosolized with electrospray ionization (13), a “soft” and commonly used ionization technique used for native mass spectrometry that is also compatible with SPI (22,34). We first thermalized the system using Born-Oppenheimer MD, employing the Nosé thermostat to conserve a temperature of 300 K. For this step of the simulation, we used a double-Z basis set with one polarization orbital per atom. The basis functions were generated with a shallow confinement potential of 0.001 Ry to allow for sufficient diffuse functions. The exchange-correlation integration grid was determined by a 200 Ry cutoff and was treated according to the van der Waals function described by Vydrov and van Voorhis (35). The thermalization simulation was 2 ps long, with a time step of 0.5 fs. Next, we oriented the protein so that its dipole moment was aligned against the external EF. Without allowing for any nuclei dynamics, we then exposed the protein to EFs ranging from 0.5 to 50.0 V/nm. For these simulations, we extended the basis set to encompass the charges in the electron distribution with respect to the ground state and used a triple-Z basis set with double polarization orbitals. For accurate partial charges, the integration mesh cutoff was increased to 500 Ry.

### Classical MD simulations

A set of gas-phase classical MD simulations were performed to study the orientation of ubiquitin exposed to a time-dependent EF. The Gromacs 4.5.7 (36) simulation package was used together with the OPLS-AA force field (37) in accordance with our previous studies of protein in gas phase (1,8,13,14,38). To sample sufficient statistics for our analysis and better mimic the heterogeneity of gas-phase experimental samples, we performed independent sets of simulations starting from different protein structures. For this purpose, starting from coordinates based on crystallographic data of ubiquitin (Protein Data Bank: 1UBQ) (39), we ran a 10 ns presimulation in solution in the NPT ensemble with Berendsen (40) weak coupling. Temperature was set to 300 K with 0.1 ps time coupling, and pressure was set to 1 bar with a time coupling of 20 ps. The TIP4P (41) water model was used. From the equilibrated portion of this bulk simulation (2.5–10 ns), we extracted structures at randomly picked times.

**Figure 1:**
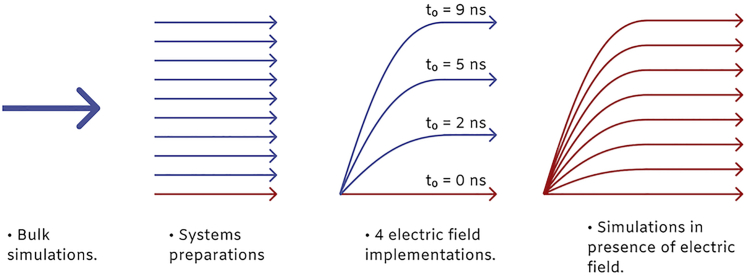
Classical simulations. The schematic representation of performed classical MD simulations is shown. For more detailed description, see Fig. S1.

The strategy we used then, depicted in Figs. 1 and S1, is the following. After removing the solvent, we assigned the protonation states of ubiquitin in vacuum according to published data (13,42,43), resulting in total charge of +7 *e*, corresponding to electrosprayed ubiquitin in its native fold (15). The systems were relaxed in vacuum, and the temperature was adjusted over 100 ps simulation to 300 K using the Berendsen thermostat (40). We then ran a 10-ns-long simulation in which we allowed the structures to equilibrate without thermostat. At the end of these pre-runs, the temperature of all the replicas was spanning a range of 305 ± 5 K. Subsequently, we performed again 100 ps simulation with a temperature coupling at 300 K to ensure all the structures were at the same temperature. The structures obtained in this way were oriented to have their dipole moment parallel to the *z* axis of the simulation box and were used as starting structures to perform the EF orientation simulations. The time-dependent EF (44) was implemented as

(1) $$E\left(t\right)=E_{0}\text{exp}\frac{-{\left(t-t_{0}\right)}^{2}}{2\sigma^{2}}\times H\left(t_{0}-t\right)+E_{0}\times H\left(t-t_{0}\right),$$

where *H*(*t*) is the Heaviside function, *t*_0_ ∈ [0, 2, 5, 9] ns and *E*_0_ ∈ [0.1, 0.2, 0.5, 0.8, 1.0, 1.5, 2.5, 3.0] V/nm. The direction of the EF was set to be parallel to the *x* axis of the simulation box. For the simulations in which *t*_0_ ∈ [0, 2, 5] ns, the duration was set to 10 ns and to 14 ns for simulations in which *t*_0_ = 9 ns. In total, 320 independent simulations were performed (10 starting structures, four choices of *t*_0_-value, eight EF strengths).

Long-range electrostatic forces in vacuum were captured using no cutoffs for nonbonded interactions. The equations of motion were propagated using the leap-frog integration scheme (45) with a 0.5 fs time step. To reproduce perfect vacuum, neither pressure coupling nor periodic boundary conditions were applied.

## Results and discussion

### Ab initio MD simulations: validation of classical MD approach

Ab initio calculations were performed on the Trp-cage protein. Given the extreme computational effort needed for quantum calculations, we limited the extend of those simulations to comprise only the electronic response, without nuclei dynamics. The main goal of these simulations is to have a quantitative estimation of the order of magnitude of the EF strength needed to break interatomic bonds, as well as validating the use of fixed charges in the classical MD simulations.

In Table 1, some of the most representative equilibrium bond forces within proteins are listed. These values were computed by dividing the tabulated bond energies by the tabulated equilibrium experimental distances. We qualitatively assume that a bond among two atoms is broken when their distance is increased by 25% with respect to their equilibrium distance. We thus define a bond dissociation force (*BDF*_*thld*_) equal to 1 eV/Å as a reasonable lower estimation of the force sufficient to break a covalent bond. This value corresponds to ∼80% of the force required to separate two sulfur atoms in a disulfide bond (Table 1).

**Table 1:** Covalent and hydrogen bond forces at the equilibrium of particular relevance in proteins

Covalent bonds (46)		Hydrogen bonds (47)	
Type	Force (eV/Å)	Type	Force (eV/Å)
C-N	~2.0	N-H⋅⋅⋅O	~0.08
C-C	~2.2	C-H⋅⋅⋅N	~0.12
C-S	~1.4	O-H⋅⋅⋅O	~0.10
C-O	~2.5	C-H⋅⋅⋅O	~0.20
S-S	~1.3	–	–

Force values are computed by dividing the tabulated energies by the tabulated equilibrium bond distances. Hydrogen bonds are indicated by ⋅⋅⋅.

We assume that the force acting on any atom *i* in our simulations can be expressed as a sum of three terms, given as

(2) $$F_{i,TOT}\left(E\right)=F\left(d_{CM}\right)+F\left(d_{i}\right)+F_{i,field}\left(E\right).$$

Here, *F*(*d*_*CM*_) is the force depending on the motion of the center of mass of the protein, *F*(*d*_*i*_) is the force due to the atomic vibrational state, and *F*_*i*,*field*_(*E*) is the contribution to the force given by the interaction with the external EF. For each simulation at the different EF strengths, we calculate the average relative force $\left\langle \left|F\left(E_{j}\right)\right|\right\rangle$ as

(3) $$\left\langle \left|F\left(E_{j}\right)\right|\right\rangle =\frac{1}{N}\overset{N}{\underset{i}{\sum }}\left|F_{i}\left(E_{j}\right)\right|-\left|F_{i}\left(E=0\right)\right|=\frac{1}{N}\overset{N}{\underset{i}{\sum }}\left|F_{i,field}\left(E\right)\right|.$$

**Figure 2:**
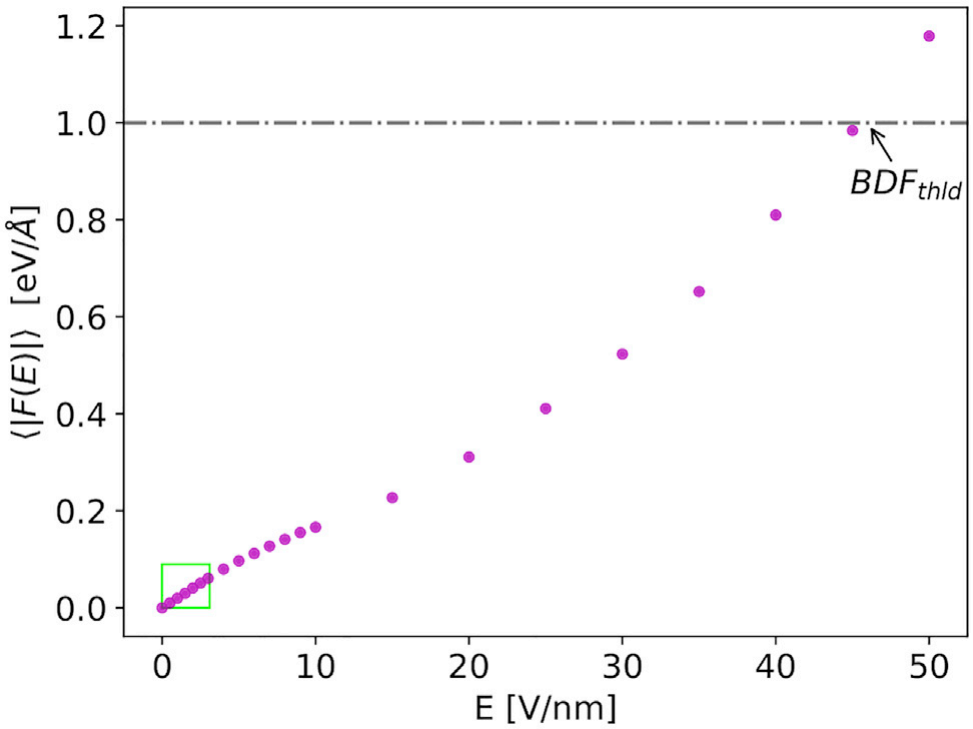
Ab initio simulations. Mean value of the force exerted on Tpr-cage atoms, defined in Eq. 3, is shown as a function of the electric field strength. The region of interest for the classical MD simulations is presented in the inset.

With this quantity, we isolate the effects induced only by presence of the external EF from the total force at a given *E*_*j*_. The contributions due to the center-of-mass motion and the particular vibrational state in which the atoms are frozen in are automatically removed. By comparing $\left\langle \left|F\left(E_{j}\right)\right|\right\rangle$ to *BDF*_*thld*_, it is possible to infer an estimation of the field strength necessary to break a covalent bond. In Fig. 2, we display $\left\langle \left|F\left(E_{j}\right)\right|\right\rangle$ as function of the simulated EF. The plot shows that the EF strength needed to break atomic bonds is of the order of 45 V/nm. Moreover, one can notice that for all the field strengths used in the classical MD (in the *green inset* of the *graph*), the value of the average force $\left\langle \left|F\left(E_{j}\right)\right|\right\rangle$ is one order of magnitude lower than *BDF*_*thld*_. This finding proves that the integrity of the protein topology is maintained in that field range and that the accuracy of classical MD is preserved.

**Figure 3:**
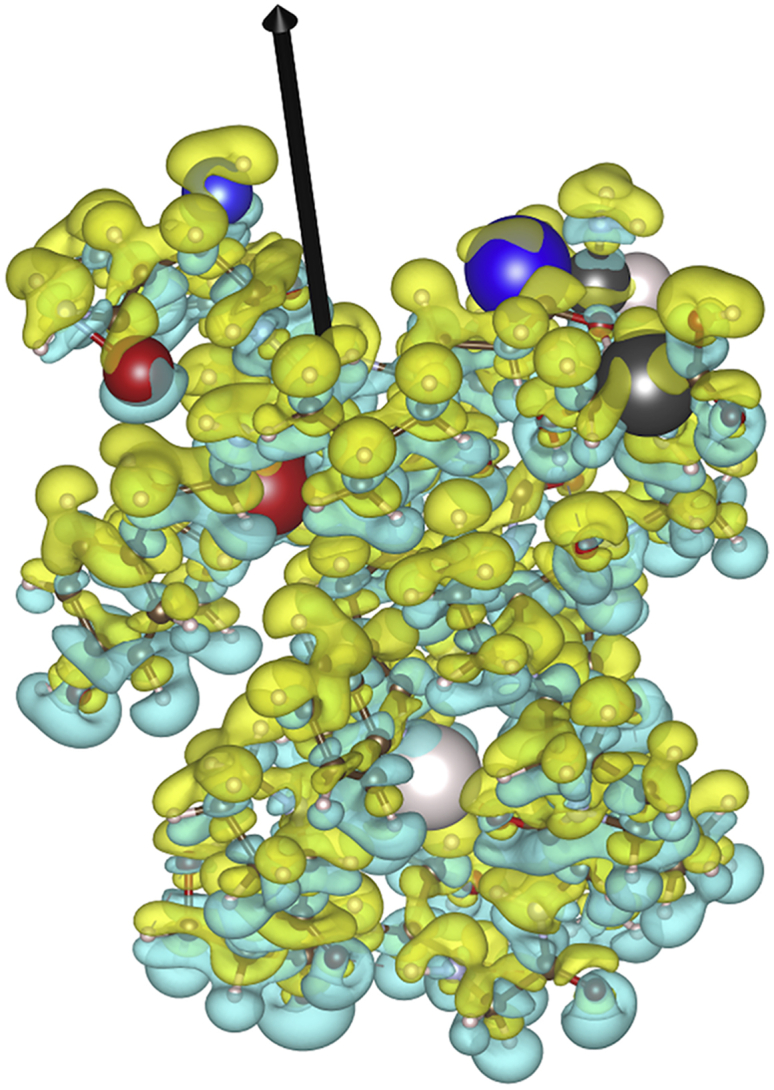
Ab initio simulations. The effect of the electric field on the electron distribution in the Trp-cage protein is shown. Displayed is the difference in electron density between a protein unexposed to the field and a protein experiencing a 3 V/nm field. Blue electron density in the figure symbolizes a loss of electron density and green an increase in electron density. Hydrogen atoms are white, oxygen red, nitrogen blue, and carbon black. Atoms that correspond to a maximal decrease of the integrate electron number difference are depicted with atomic radii increased by a factor of 2; atoms that correspond to a maximal increase of the integrate electron number difference are depicted with atomic radii enlarged by a factor of 3. The isosurface level is set to 0.00226 electrons/Å^3^. The black arrow denotes the direction of the external electric field.

In Fig. 3, the polarization induced by 3.0 V/nm field on the electron density is shown. At this field strength, the majority of the polarization response is local to the atoms. Hence, not much charge is transferred across the molecule, and the maximal charge increase and decrease in relation to the ground state of a specific atom are relatively small. This validates the approach used in our classical MD simulations, in which the ionic charge is fixed throughout the simulation. In Table 2, we list the maximal increase and decrease in integrated charge on the atoms for which the difference is largest. The corresponding positions of the respective atoms are shown in Fig. 3. The fact that these atoms are not residing at the edge of the protein also indicates the importance of the local polarization above delocalized charge transfer across the molecule, further corroborating the use of classical MD in this context.

**Table 2:** The maximal integrated electron number difference around each site for each atomic species

Atom type	Maximal charge increase	Maximal charge decrease
Hydrogen	0.016	−0.018
Carbon	0.022	−0.013
Nitrogen	0.012	−0.017
Oxygen	0.010	−0.014

Units are in electronic charge *e*. The integrated difference is defined as “number of electrons on site X at field = 0” − “number of electrons on site X with field = 3 V/nm.” The corresponding atoms are highlighted in Fig. 3 by enlarging their radii by a factor of 2 for the maximal decrease and a factor of 3 for the maximal increase.

### Classical MD simulations: orientation in time-dependent EFs

The way the protein responds to the time-dependent EF has been assessed by studying three different observables: degree of orientation, speed of orientation, and the root mean-square deviation (RMSD) of the atomic positions. First of all, to assess the extent of protein orientation we define the degree of orientation as

(4) Θ=1−cos(θ),

where *θ* is the angle between the EF and the total dipole moment of the molecule. Thus, a fully aligned protein expresses a value of Θ = 0, whereas Θ = 1 corresponds to a perpendicular orientation when considering a protein at a particular moment in time. Θ = 1 is, however, also the expectation value for a randomly oriented protein, as parallel and antiparallel orientations then are equally likely and the average cos(*θ*) becomes 0.

**Figure 4:**
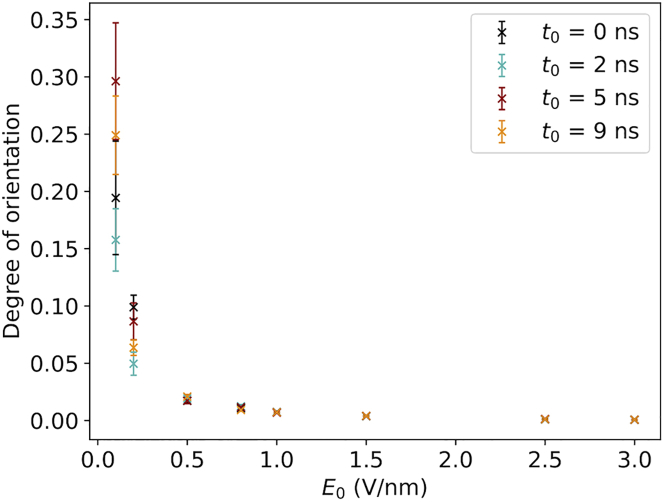
Classical simulations. An averaged degree of orientation is shown as a function of the maximal field strength *E*_0_ for different ramp-up times *t*_0_. The different colors refer to the different field implementations (*black*, *t*_0_ = 0 ns; *cyan*, *t*_0_ = 2 ns; *red*, *t*_0_ = 5 ns; and *orange*, *t*_0_ = 9 ns).

In Fig. 4, we show how Θ depends on the EF strength *E*_0_ and the ramping time *t*_0_. Values in Fig. 4 show the average degree of orientation over the last 2 ns of the 10 independent simulations. As one could expect, the stronger the field is, the more the molecule becomes oriented. In Fig. S2, the projection of the dipole moment of the plane perpendicular to the EF vector is depicted. Here, an *E*_0_ of 0.1 V/nm is not enough to orient the protein in the simulation time we explored, and the projection of the dipole moment is equally distributed in the plane, regardless of the value of *t*_0_. For *E*_0_ equal to 0.2 V/nm, although there is not perfect alignment of the EF and the dipole of the molecule, the dipole moment distribution is not completely random. In particular, quite interestingly, the EF implementation with a ramping-up time equal to 2 ns results in a better orientation respect to the other three EF implementations; although not very focused, one can notice a highly populated region corresponding to a spread of ±15° with respect to perfect alignment. For field values equal to 0.5 V/nm, the projection of the dipole moment in plane perpendicular to the field is focused in the ±15° region, expressing a good alignment between the field vector and the ubiquitin dipole moment. For field strengths greater than or equal to 0.5 V/nm, the differences in the degree of orientation among the ramping times are not resolved within the errors (Figs. 4, S3, and S4). At the end of the simulations, the protein is oriented in a similar way, regardless of the field implementation.

**Figure 5:**
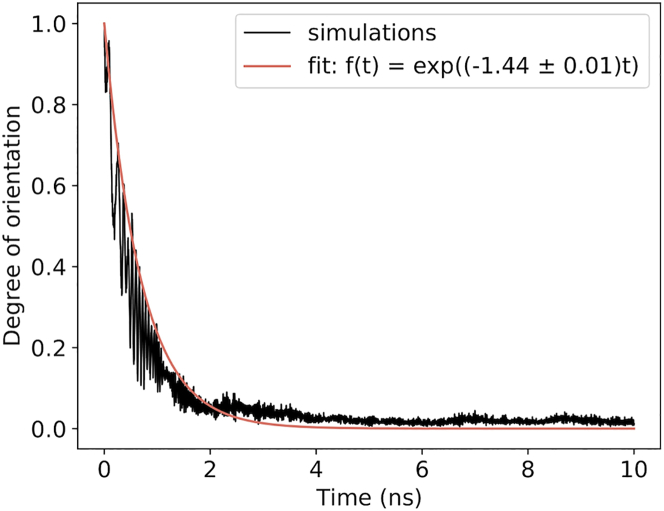
Classical simulations. The time evolution of degree of orientation averaged over 10 independent runs for the parameters *E*_0_ = 0.5 V/nm, *t*_0_ = 2 ns (*black line*) is shown. The orange line is the result of fitting it with the function *f*(*t*) = exp(−*kt*).

The second observable we monitored in our simulations was the speed of orientation. Although the degree of orientation oscillates significantly, we can observe that there is a clear exponential decay. In Fig. 5, an example of this trend for *E*_0_ = 0.5 V/nm, *t*_0_ = 2 ns is presented. Here, the black line represents the evolution of $\bar{\text{Θ}}$ over time $\left(\bar{\text{Θ}\left(t\right)}\equiv \langle \text{Θ}\left(t\right)\rangle_{replicas}\right)$. The orange line represents a fit on $\bar{\text{Θ}}$(*t*), with *f*(*t*) = exp(−*kt*). We define *τ* as the time required for the protein to lose 90% of its initial orientation and hence to arrange near parallel to the EF vector:

(5) $$\tau =\frac{\text{ln}\left(10\right)}{k}.$$

**Figure 6:**
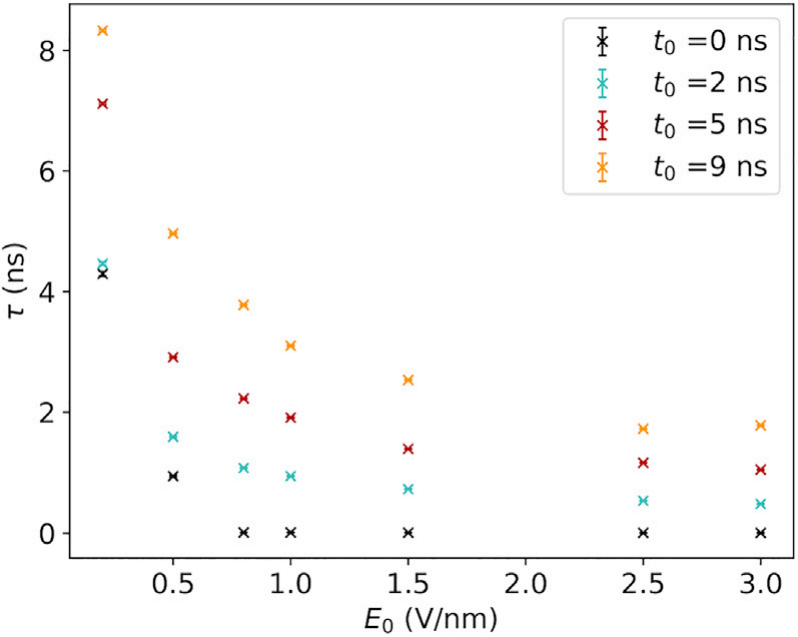
Classical simulations. The dependence of time *τ* (when the protein lost 90% of initial orientation) on *E*_0_ for different ramping time *t*_0_ is shown. The color scheme is the same one described in Fig. 4.

In Fig. 6, we display the correlation between the orientation time *τ* for all the simulated field implementations *t*_0_ and *E*_0_. Evidently, a clear dependence between the rate of orientation and the ramping time can be observed: the longer the ramping time is, the more time is needed to orient the structure. Here, *τ* spans a range of values from 8.3 ns for *E*_0_ = 0.2 V/nm and *t*_0_ = 14 ns to 3 ps for *E*_0_ = 3.0 V/nm and *t*_0_ = 0 ns.

**Figure 7:**
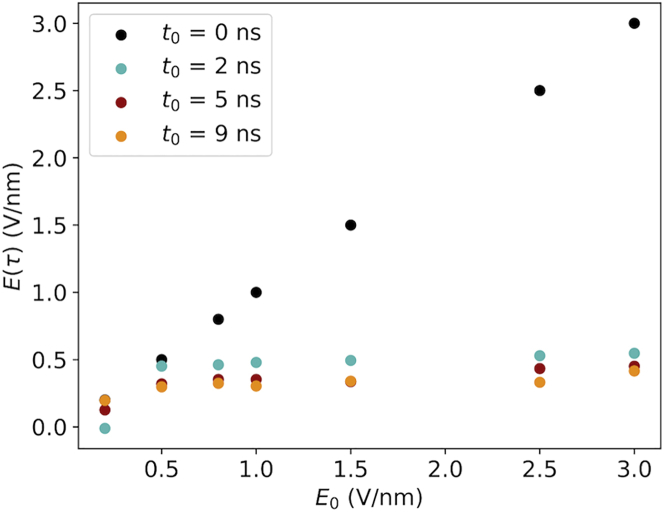
Classical simulations. EF strength at the time the protein is oriented is shown as a function of the EF implementation. The color scheme is the same described in Fig. 4.

The question naturally arises: is there any particular field strength able to orient the protein? To answer this query, we plot, in Fig. 7, *E*(*τ*)(*E*_0_;*t*_0_), namely the value of the EF strength at the time the protein is oriented as a function of the field implementations for the different simulations. Notably, except for the trivial case of the constant field (*t*_0_ = 0 ns), the field strength required to align the protein according to this strict orientation criterion seems to be always on the order of 0.5 V/nm, independently of the final field strength of the simulation and the implementations. In other words, an EF strength of 0.5 V/nm is a necessary and sufficient condition to achieve a strong alignment of ubiquitin within 10 ns.

The last important question we assessed regards the structural stability. It is crucial to consider the possible structural changes induced by the presence of an external EF. RMSD computed on C*α* atoms gives a measure of how much the structure has changed relative an earlier time point. It is reasonable to define a structure to be preserved if the RMSD value is below 0.5 nm (1), whereas for RMSD values higher than this threshold, we can assume that the protein’s initial structure is lost.

**Figure 8:**
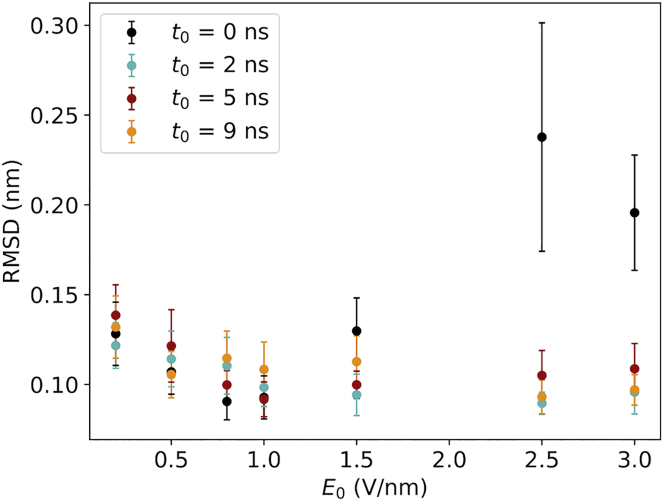
Classical simulations. The dependence of RMSD at time *t* = *τ* on *E*_0_ for different ramping time *t*_0_ is shown. The color scheme is the same one described in Fig. 4.

**Figure 9:**
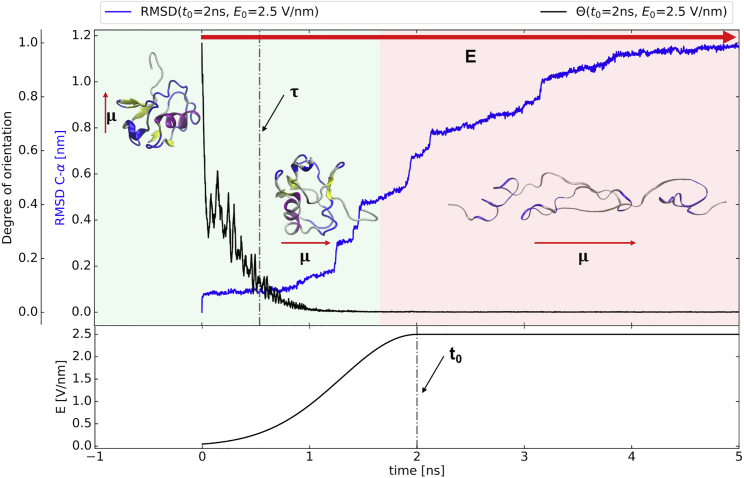
Classical simulations. Time evolution of the degree of orientation, RMSD and electric field function for *t*_0_ = 2 ns and *E*_0_ = 2.5 V/nm simulations is shown. Only the first 5 ns are shown. Each data point shows the result of the averaged values of the 10 replicas. Error bars are not shown for simplicity. The green background represents the part of the simulation in which the ubiquitin structure is preserved (RMSD ≤ 0.5 nm). On the contrary, the red background indicates the part of the simulation in which the protein structure is lost. The red arrow denoted with *E* represents the direction of the external EF; with *μ* arrows, we represent the direction of the protein dipole. In the insets, cartoon representations of the protein structure at the corresponding time are presented.

What is the state of the structures when the proteins become oriented by the field? In Fig. 8, RMSD(*τ*) (namely the RMSD value at time *t* = *τ*) as a function of *t*_0_ and *E*_0_ are displayed for the different field implementations. We can observe that all ramping-up times provide good conservation of the protein structures at the time they are oriented. This assumption is valid for all the values of the EF we tested. We can therefore conclude that in all cases we simulated, the orientation happens before the structure is damaged. An example of this order of events is visualized in Fig. 9.

**Figure 10:**
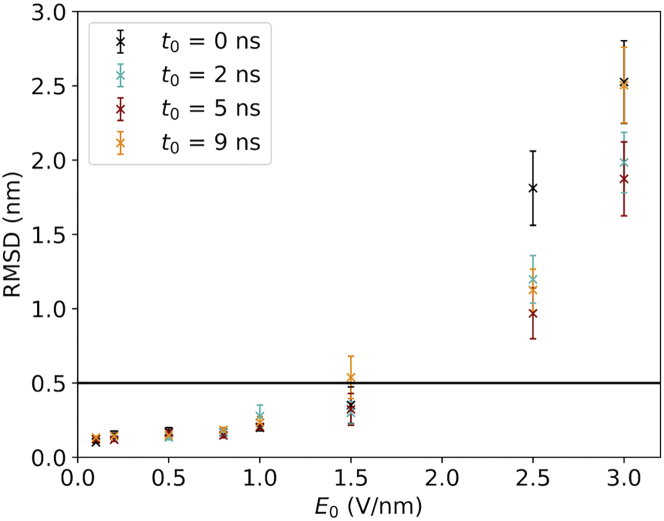
Classical simulations. Dependence of RMSD after 5 ns of EF reached its maximal value is shown as a function of *E*_0_ and *t*_0_. The color scheme is the same one described in Fig. 4.

Lastly, we ask how the protein structures evolve after the EF has reached its maximal strength. To this end, we evaluated the RMSD values of ubiquitin after 5 ns after *t*_0_, that is, we included the trajectory data starting at 5 ns from the beginning of the simulations for the case of *t*_0_ = 0 ns, 7 ns for *t*_0_ = 2 ns, 10 ns for *t*_0_ = 5 ns, and lastly 14 ns for the case of *t*_0_ = 9 ns. In SPI, this captures the possibility to acquire the image of the intact protein using the x-ray beam after the molecule traveled in the experimental device. The results of this analysis are presented in Fig. 10, in which for each data point, an average over the 10 replicas was computed. One can observe for EF strengths lower than or equal to 1.5 V/nm, the protein structures are maintained for all the EF implementations. In particular, for an EF lower than or equal to 0.8 V/nm, there are almost no differences among the four different EF implementations. In contrast, EF strengths greater than or equal to 2.5 V/nm yield RMSD values above the 0.5 nm threshold for all the EF implementations, indicating that the structures are no longer native like.

## Conclusions

We have investigated the controlled orientation of proteins in the gas phase using time-dependent external EFs. Time-varying EFs is an inescapable aspect of physical reality of any experimental setup for field orientation, as the proteins must either enter the high-field region from a low-field region or be exposed to a pulsed field that necessarily has a smooth temporal profile at fast timescales, in both cases experiencing an EF that increases over time. As such, this investigation considers an inherent aspect of orientation control of protein using EFs that has been disregarded from earlier studies. Moreover, the pulse profile could, in principle, affect the response of the protein to the applied EF, both in terms of structural damage and orientation, and is an important experimental parameter to consider also for that reason. Our results show that the temporal profile makes little difference for the degree of orientation after 10 ns (Fig. 4). The orientation time *τ* when the protein is 90% oriented, however, displays a dependence on ramp-up time *t*_0_, which is not unexpected given that higher *t*_0_ implies that the protein will start experiencing a strong field at a later time. More surprisingly, the EF strength at *t* = *τ* (*E*(*τ*)) appears to be independent of *t*_0_ and takes a value of ∼0.5 V/nm (Fig. 7). We note that this value is comparable with but nonetheless higher than the EF strengths used by Hekstra et al. (3) for inducing motions in protein crystals. We have imposed a rather strict criterion, however, and more relaxed criteria would yield lower values for *E*(*τ*). Orientation of proteins using EFs has been shown to benefit orientation recovery in SPI, but how strong a control is needed for it to be useful is still unknown and an interesting topic for future research. Applications in which only a slight bias is needed will more readily exploit the phenomenon investigated herein, as the lower required field strengths pose less of a challenge to produce. It should be emphasized that using EFs to orient proteins does not provide control over all rotational degrees of freedom. Even for Θ approaching 0, corresponding to perfect alignment with the EF, the protein will be free to rotate around the EF vector. Consequently, EFs cannot be used with applications that require full control over all 3D rotations.

In our investigation, we did not consider residual water molecules on the proteins explicitly. First, the experimental setup might not allow for retained water, but even if it did, their contribution to the net dipole moment would be small. First, only a small number (∼10–15) of water molecules are strongly bound to the structure (38). The dipole moment of a single water molecule is furthermore 1.85 D, so even if all water molecules were perfectly aligned, their collective contribution would be small compared to the net dipole of ubiquitin. The water could affect the stability of the protein, but previous investigations have indicated a preserving, and not a destructive, effect (13,14).

We used classical MD to study orientation with EFs. To account for the possibility of bond breaking because of the EF, we carried out quantum-mechanics simulations, which enabled us to identify an approximate limit at 45 V/nm corresponding to the *BDF*_*thld*_, below which bonds remain intact. This value is considerably higher than what has been used in this context before (1,8) and in this study. Importantly, because the interactions that make up protein structures are chiefly the same for all proteins, we can expect our estimate to not be very system dependent. Even without bond breaking, however, the EF could complicate any classical modeling by displacing electrons across the protein, but we found that electrons only shift locally (Fig. 3). Because the electrons remain largely in place and we only considered EF strengths at least an order of magnitude below the bond-breaking limit, we could safely use classical MD for our investigation. The main remaining form of damage that the EFs can cause to the structure of a protein comes from the opposite forces acting on (partial) charges of opposite signs, which can break the noncovalent interactions that keep the fold intact. This happens with very high static EFs, but surprisingly, for nonzero values of *t*_0_, the RMSD of ubiquitin remained low at the moment when the protein had become 90% oriented, meaning that the damage induced by the EF builds up slower than the orientation time of the protein. This feature of “orientation before destruction” can be exploited to probe oriented proteins with unperturbed structures if the time between the EF pulse and the measurement (such as the x-ray pulse in SPI) can be tightly controlled, even for destructive EF strengths.

In our previous work, we noted that longer exposure times also reduced the EF strength needed for orientation, but also for structural loss (1), which entails that the time a protein spends in the field can be expected to modulate the trends we have found here. We also note that we have used a relatively small globular protein as a model system because it is more readily simulated, whereas the systems imaged with SPI so far have been considerably larger. The scattering power scales with the mass of the particle exposed to the beam, and large macromolecular complexes simply give a stronger signal and better signal/noise ratio, making them easier to image. Serendipitously, the expected dipole moment of proteins also increases with protein mass (2), making for a stronger interaction with the EF and potentially making large protein complexes orient more readily in the EF. How this combines with the increase in moment of inertia for larger proteins remains to be seen, but in our earlier work, we could discern a trend in which in the lower end of the investigated range of EF strengths, the larger of the proteins we simulated were more oriented. We therefore speculate that for proteins that are amenable for SPI, *E*(*τ*) will take on lower values, especially if longer exposure times are used, matching the EFs that have already been used in other applications (3,31).

## Author contributions

C.C., E.G.M., and E.D.S. devised the project. C.C., with help from O.G., performed ab initio simulations. H.A., T.M., and A.S. carried out classical simulations. A.S. and E.D.S. analyzed the data. E.D.S., with help from C.C., O.G., E.G.M., and A.S., wrote the article.

## Supporting material

Document S1. Figs. S1–S4 and Table S1

Document S2. Article plus supporting material
