# Supplementary material for: Protein orientation in time-dependent electric fields: orientation before destruction
Source: Biophys J. 2021 Jul 23;120(17):3709–17. doi: 10.1016/j.bpj.2021.07.017 (PMC8456286; doi:10.1016/j.bpj.2021.07.017)
Supplement: Document S1. Figs. S1–S4 and Table S1 [file mmc1.pdf]

Biophysical Journal, Volume *120*

Supplemental information

Protein orientation in time-dependent electric fields: orientation before destruction

Anna Sinelnikova, Thomas Mandl, Harald Agelii, Oscar Grånäs, Erik G. Marklund, Carl Caleman, and Emiliano De Santis

Manuscript submitted to **Biophysical** *Journal*
Article

Supporting Material:
Protein orientation in time-dependent electric fields: Orientation before destruction

A Sinelnikova[1], T Mandl[1,2], H Agelii[1], O Grånäs[1], E G Marklund[3], C Caleman[1,4], E De Santis[1,3,*], Anna Sinelnikova[1], Thomas Mandl[1,2], Harald Agelii[1], Oscar Grånäs[1], Erik G. Marklund[3], Carl Caleman[1,4], and Emiliano De Santis[1,3,*]

[1]Department of Physics and Astronomy, Uppsala University, Box 516, SE-751 20 Uppsala, Sweden
[2]University of Applied Sciences Technikum Wien, Höchstädtplatz 6, A-1200 Wien, Austria
[3]Department of Chemistry – BMC, Uppsala University, Box 576, SE-751 23 Uppsala, Sweden
[4]Center for Free-Electron Laser Science, DESY, Notkestrasse 85, DE-22607 Hamburg, Germany
[*]Correspondence: emiliano.desantis@physics.uu.se

ab initio MD simulations

Table S1: Average relative force, $\langle|F(E)|\rangle$, as a function of the electric field strength. Values are given in eV/Å units.

**Electric field**	$\langle|F(E)|\rangle$
0.0	0.000
0.5	0.010
1.0	0.020
1.5	0.030
2.0	0.041
2.5	0.051
3.0	0.061
4.0	0.080
5.0	0.097
6.0	0.112
7.0	0.127
8.0	0.141
9.0	0.155
10.0	0.166
20.0	0.311
25.0	0.411
30.0	0.523
35.0	0.652
40.0	0.810
45.0	0.984
50.0	1.179

Classical MD simulations

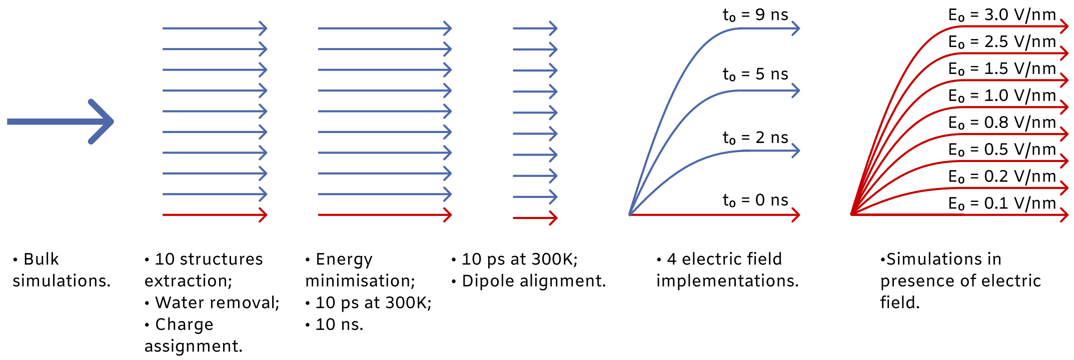

Figure S1: The schematic representation of performed classical MD simulations.

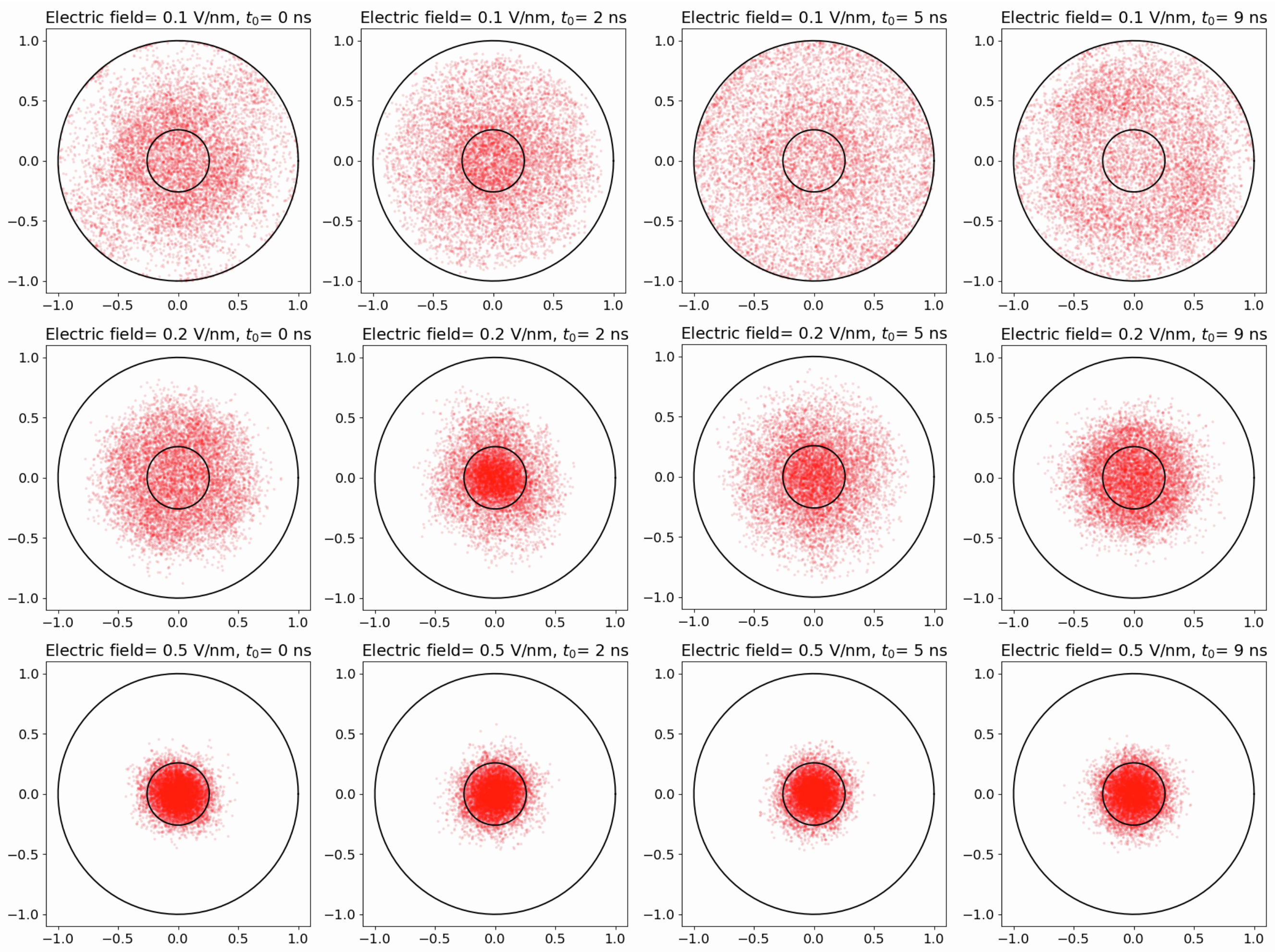

Figure S2: Scatter plot of the projection of the dipole moment on the yz plane for the simulations with electric field strength of 0.1 V/nm (first row), 0.2 V/nm (middle row) and 0.5 V/nm (lower row) . Each point of every panel refers to a frame taken in the last 2 ns of simulations for all the ten replicas. Each panel contains 8000 points (the frames are taken every 2.5 ps of simulations). The circle drawn in the center of the panels represent an uncertainty of ± 15 degrees respect to a perfect alignment.

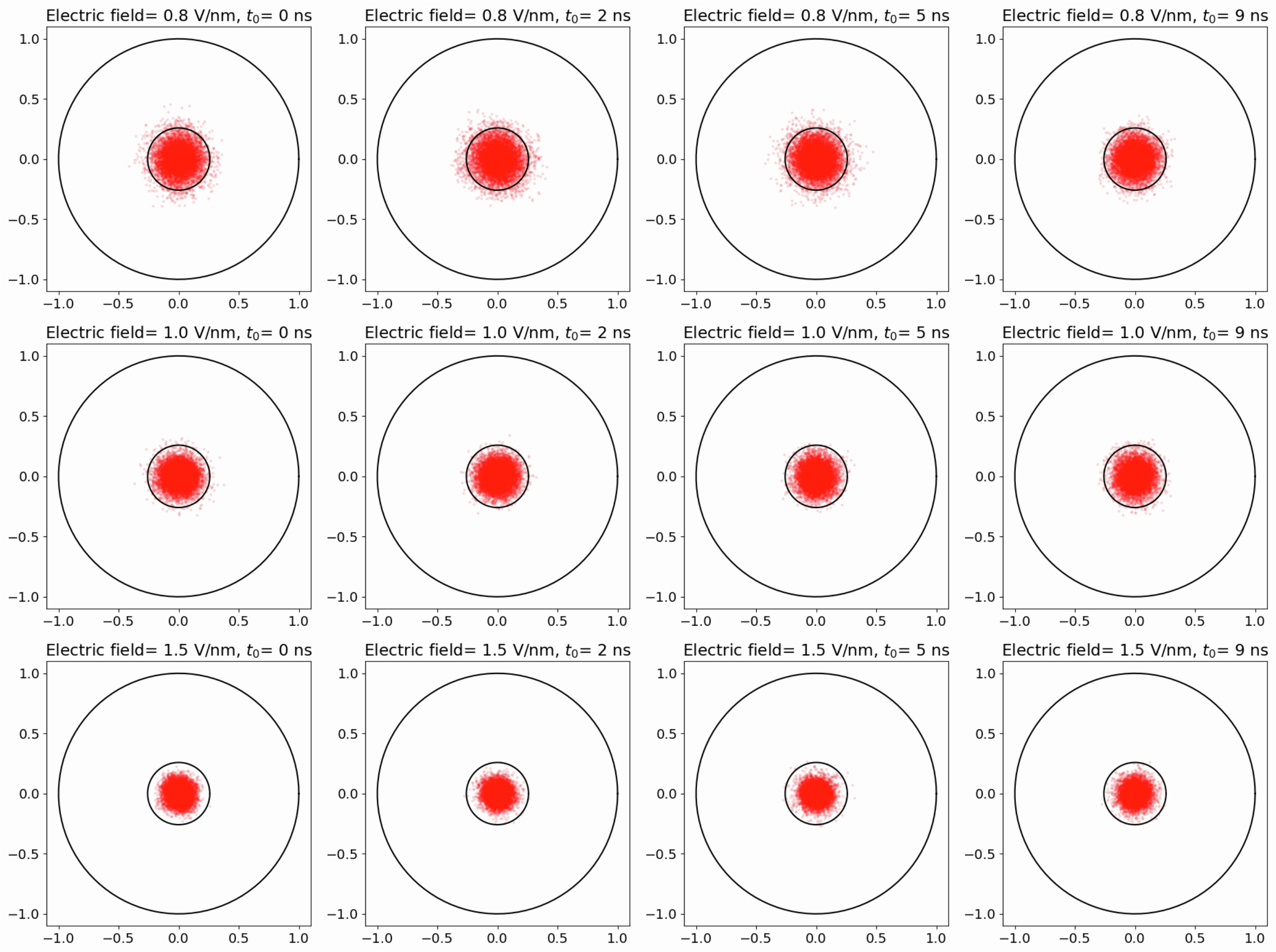

Figure S3: Scatter plot of the projection of the dipole moment on the yz plane for the simulations with electric field strength of 0.8 V/nm (first row), 1.0 V/nm (middle row) and 1.5 V/nm (lower row) . Each point of every panel refers to a frame taken in the last 2 ns of simulations for all the ten replicas. Each panel contains 8000 points (the frames are taken every 2.5 ps of simulations). The circle drawn in the center of the panels represent an uncertainty of ± 15 degrees respect to a perfect alignment.

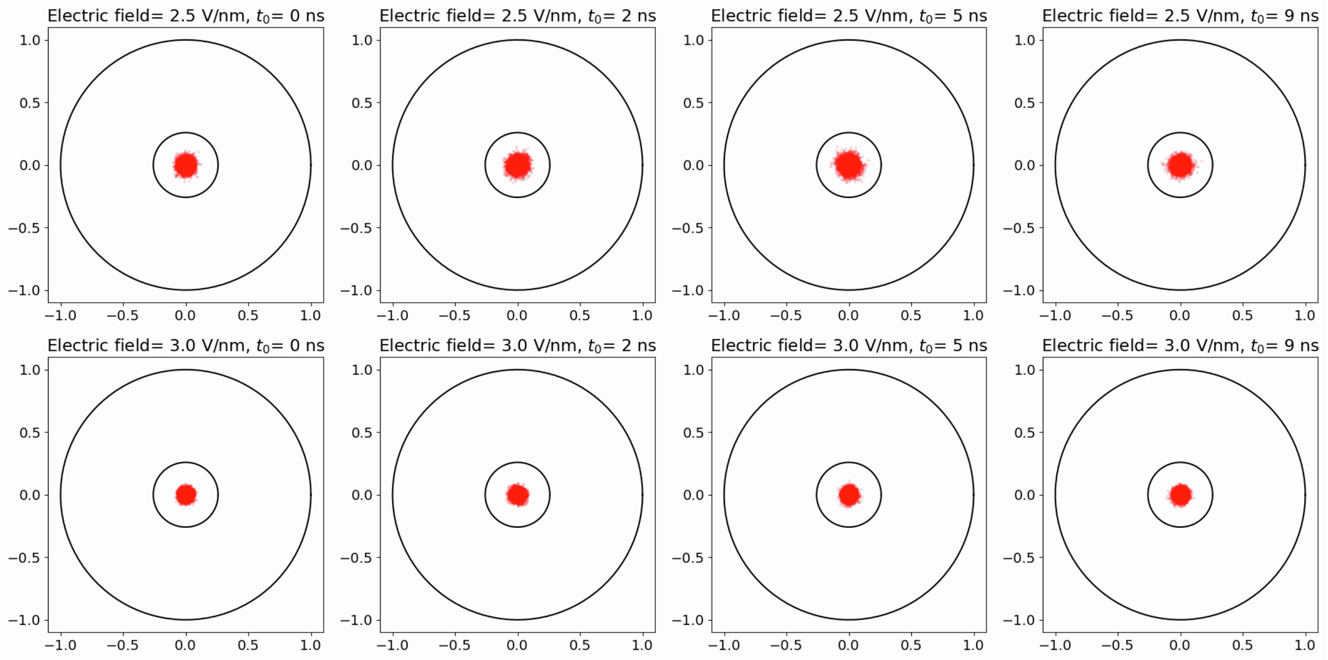

Figure S4: Scatter plot of the projection of the dipole moment on the yz plane for the simulations with electric field strength of 2.5 V/nm (first row) and 3.0 V/nm (lower row) . Each point of every panel refers to a frame taken in the last 2 ns of simulations for all the ten replicas. Each panel contains 8000 points (the frames are taken every 2.5 ps of simulations). The circle drawn in the center of the panels represent an uncertainty of ± 15 degrees respect to a perfect alignment.
