# Supplementary material for: Protein orientation in time-dependent electric fields: orientation before destruction
Source: Biophys J. 2021 Jul 23;120(17):3709–17. doi: 10.1016/j.bpj.2021.07.017 (PMC8456286; doi:10.1016/j.bpj.2021.07.017)
Supplement: Document S2. Article plus supporting material [file mmc2.pdf]

Biophysical*Journal*
Article

Biophysical Society

Protein orientation in time-dependent electric fields: orientation before destruction

Anna Sinelnikova,[1] Thomas Mandl,[1,2] Harald Agelii,[1] Oscar Grånäs,[1] Erik G. Marklund,[3] Carl Caleman,[1,4] and Emiliano De Santis[1,3,*]

[1]Department of Physics and Astronomy, Uppsala University, Uppsala, Sweden; [2]University of Applied Sciences Technikum Wien, Wien, Austria; [3]Department of Chemistry BMC, Uppsala University, Uppsala, Sweden; and [4]Center for Free-Electron Laser Science, DESY, Hamburg, Germany

ABSTRACT Proteins often have nonzero electric dipole moments, making them interact with external electric fields and offering a means for controlling their orientation. One application that is known to benefit from orientation control is single-particle imaging with x-ray free-electron lasers, in which diffraction is recorded from proteins in the gas phase to determine their structures. To this point, theoretical investigations into this phenomenon have assumed that the field experienced by the proteins is constant or a perfect step function, whereas any real-world pulse will be smooth. Here, we explore the possibility of orienting gas-phase proteins using time-dependent electric fields. We performed ab initio simulations to estimate the field strength required to break protein bonds, with 45 V/nm as a breaking point value. We then simulated ubiquitin in time-dependent electric fields using classical molecular dynamics. The minimal field strength required for orientation within 10 ns was on the order of 0.5 V/nm. Although high fields can be destructive for the structure, the structures in our simulations were preserved until orientation was achieved regardless of field strength, a principle we denote "orientation before destruction."

SIGNIFICANCE New means for controlling molecules enable new science and applications. Recent investigations show that the orientation of proteins can be controlled with electric fields in vacuum, but the fields have been assumed to be turned on instantaneously, which is a poor description of the field experienced by a protein in a laboratory setting. Here, we instead explore the possibility of orienting proteins with time-dependent electric fields, using quantum-mechanics calculations to test the integrity of covalent bonds and classical simulations to monitor orientation and preservation of the protein structures. Our results advance our understanding of the process of dipole orientation and provide a more realistic picture upon which to base the design of an experimental apparatus to take this phenomenon from theory to practice.

INTRODUCTION

New means for manipulating macromolecules can be of great utility for both applications and basic research in the physical and life sciences. Using classical molecular dynamics (MD) simulations, we recently demonstrated the feasibility of controlling the orientation of gas-phase proteins using a strong electric field (EF) (1). Proteins often carry an electric dipole moment (2), which interacts with the EF to generate a torque on the protein. Using EFs to manipulate proteins is not novel per se; strong EFs were used to cause domain motions in crystals of proteins in x-ray crystallography (3), and EF interactions underpin numerous separation techniques in both solution and gas phase. The field can be destructive for the protein structures, as positively and negatively charged moieties will be pulled in opposite directions, potentially leading to unfolding. Deliberate unfolding can in some cases be desired because it can inform about a protein's structural and mechanical properties (4–7), and EFs can be used to this end (8), but many applications might require native or native-like structures. If an appropriate EF strength is chosen, however, the proteins orient without significant structural loss, enabling orientation control as part of protein investigations in the gas phase. Proteins are normally evolved for aqueous solutions, and the gas-phase conditions alone can be destructive for their structures. The protein backbone is, however, believed to remain folded on short timescales (9,10), which is supported by a growing mass of evidence from simulations and experiments (9,11–19). Such kinetic trapping of the structures enables delivery of intact gas-phase

Submitted February 9, 2021, and accepted for publication July 19, 2021.

*Correspondence: emiliano.desantis@physics.uu.se

Editor: Alan Grossfield.

https://doi.org/10.1016/j.bpj.2021.07.017

© 2021 Biophysical Society.

This is an open access article under the CC BY license (http://creativecommons.org/licenses/by/4.0/).

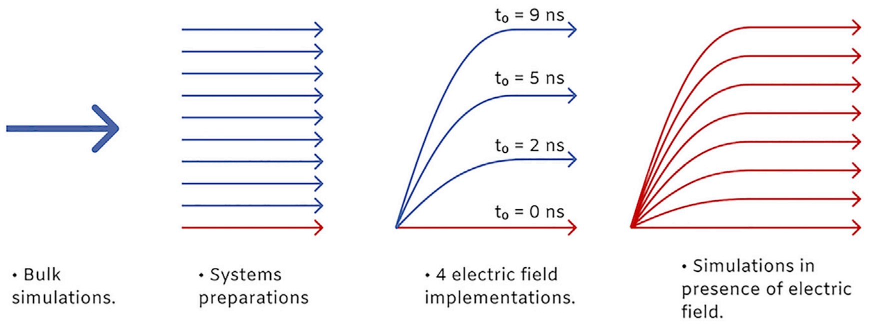

FIGURE 1 Classical simulations. The schematic representation of performed classical MD simulations is shown. For more detailed description, see Fig. S1.

proteins for ion mobility or mass spectrometry, or into an XFEL beam.

One application that benefits from orientation control of proteins is single-particle imaging (SPI; also "flash x-ray imaging") (1,20), which is a technique for structure determination in which single proteins or other nanoscale particles are exposed to x-ray pulses and the resulting diffraction is used to reconstruct the three-dimensional (3D) structure of the objects under study (21,22). Unlike x-ray crystallography, which has been the dominant technique in structural biology for decades, SPI does not require crystalline samples, which enables imaging of proteins that do not crystallize at all or do not crystallize in the states of interest, e.g., because of inherent dynamics or polydispersity. To get enough diffraction from a single protein, the pulses need to be ultraintense, which quickly obliterates the protein, and the next diffraction pattern is recorded from another identical copy of the protein. The high intensity means that the pulses also need to be ultrashort so that they scatter from the unperturbed protein structure before radiation damage has time to build up, a principle that has become known as "diffraction before destruction." Using ultrashort pulses (tens of femtoseconds) gives SPI a tremendous potential to study dynamics and kinetics in protein structures, for example, using photoexcitation to trigger a reaction just before XFEL beam exposure or fast mixing just before injection (23), in which the kinetic trapping serves to carry over the structural ensemble from solution to the gas phase. These extreme requirements are met by x-ray free-electron lasers (XFELs), such as Linac Coherent Light Source (LCLS) (24) and the European XFEL (25), which has enabled 3D imaging of the Mimi virus, revealing not only its capsid but also the genome inside, albeit at low resolution (26). Subsequent experiments have revealed the 3D structures of additional viruses (27–29) as well as carboxysomes (30).

The proteins enter the beam randomly oriented in SPI, which complicates the process of assembling the diffraction patterns into a self-consistent 3D data set—the "orientation recovery." We have shown that prior knowledge about the orientation can support the orientation recovery, even if that knowledge is incomplete (1). Importantly, it can make the orientation-recovery algorithms converge with fewer diffraction patterns and cope better with missing data. This can make the difference between successful and failed experiments, yield better structure models, and reduce sample consumption and beam time. To this point, EF orientation has only been explored assuming static fields or step functions, but any real instrument will inevitably have a smooth pulse profile (see, e.g., Wilks et al. (31)). As such, to harness field orientation in experiments, the effects of time-dependent EFs need to be investigated. Moreover, the immediate onset of the EF might not be optimal for preserving the structure, which further motivates us to here investigate how time-varying EFs perform in orienting proteins in the gas phase and what they do to the structures. We apply a multiscale approach comprising both ab initio and classical MD simulations. Using time-dependent density functional theory in the presence of an EF, we identify the upper field strength at which covalent bonds can remain intact in a protein, defining a validity limit for classical MD. We moreover performed gas-phase classical MD simulations on ubiquitin to study the effect of time-dependent EFs on its orientation and the consequent structural evolution. Our results are key for understanding the process of dipole orientation induced by an external, time-dependent EF. As such, they can serve to guide in the design of an apparatus able to control this phenomenon to manipulate proteins in SPI and other applications.

MATERIALS AND METHODS

Ab initio MD simulations

The strong EFs applied in this study create rather extreme conditions for protein molecules, and one might ask whether EF orientation can be explored using classical models. We therefore carry out ab initio MD simulations to evaluate the impact on the integrity of covalent bonds and on the overall electron distribution. A molecule becomes polarized when subjected to external EFs as the charge in the molecule rearrange to screen the EF. Consequently, this generally changes the molecular dipole. Moreover, the rearrangements of the electronic structure result in residual forces on charged sites in the protein. We used the ab initio MD software package Siesta 4.1 (32) to estimate the forces resulting from the interaction with the EF. We followed the same procedure as published in our earlier work (8). Ab initio calculations were carried out on a small protein, Trp-cage (Protein Data Bank: 1L2Y) (33), with a total charge of $+2\ e$, as is expected for Trp-cage aerosolized with electrospray ionization (13), a "soft" and commonly used ionization technique used for native mass spectrometry that is also compatible with SPI (22,34). We first thermalized the system using Born-

TABLE 1 Covalent and hydrogen bond forces at the equilibrium of particular relevance in proteins

Covalent bonds (46)		Hydrogen bonds (47)	
Type	Force (eV/Å)	Type	Force (eV/Å)
C-N	~2.0	N-H···O	~0.08
C-C	~2.2	C-H···N	~0.12
C-S	~1.4	O-H···O	~0.10
C-O	~2.5	C-H···O	~0.20
S-S	~1.3	–	–

Force values are computed by dividing the tabulated energies by the tabulated equilibrium bond distances. Hydrogen bonds are indicated by ···.

Oppenheimer MD, employing the Nosé thermostat to conserve a temperature of 300 K. For this step of the simulation, we used a double-Z basis set with one polarization orbital per atom. The basis functions were generated with a shallow confinement potential of 0.001 Ry to allow for sufficient diffuse functions. The exchange-correlation integration grid was determined by a 200 Ry cutoff and was treated according to the van der Waals function described by Vydrov and van Voorhis (35). The thermalization simulation was 2 ps long, with a time step of 0.5 fs. Next, we oriented the protein so that its dipole moment was aligned against the external EF. Without allowing for any nuclei dynamics, we then exposed the protein to EFs ranging from 0.5 to 50.0 V/nm. For these simulations, we extended the basis set to encompass the charges in the electron distribution with respect to the ground state and used a triple-Z basis set with double polarization orbitals. For accurate partial charges, the integration mesh cutoff was increased to 500 Ry.

Classical MD simulations

A set of gas-phase classical MD simulations were performed to study the orientation of ubiquitin exposed to a time-dependent EF. The Gromacs 4.5.7 (36) simulation package was used together with the OPLS-AA force field (37) in accordance with our previous studies of protein in gas phase (1,8,13,14,38). To sample sufficient statistics for our analysis and better mimic the heterogeneity of gas-phase experimental samples, we performed independent sets of simulations starting from different protein structures. For this purpose, starting from coordinates based on crystallographic data of ubiquitin (Protein Data Bank: 1UBQ) (39), we ran a 10 ns presimulation in solution in the NPT ensemble with Berendsen (40) weak coupling. Temperature was set to 300 K with 0.1 ps time coupling, and pressure was set to 1 bar with a time coupling of 20 ps. The TIP4P (41) water model was used. From the equilibrated portion of this bulk simulation (2.5–10 ns), we extracted structures at randomly picked times.

The strategy we used then, depicted in Figs. 1 and S1, is the following. After removing the solvent, we assigned the protonation states of ubiquitin in vacuum according to published data (13,42,43), resulting in total charge of $+7\ e$, corresponding to electrosprayed ubiquitin in its native fold (15). The systems were relaxed in vacuum, and the temperature was adjusted over 100 ps simulation to 300 K using the Berendsen thermostat (40). We then ran a 10-ns-long simulation in which we allowed the structures to equilibrate without thermostat. At the end of these pre-runs, the temperature of all the replicas was spanning a range of 305 ± 5 K. Subsequently, we performed again 100 ps simulation with a temperature coupling at 300 K to ensure all the structures were at the same temperature. The structures obtained in this way were oriented to have their dipole moment parallel to the z axis of the simulation box and were used as starting structures to perform the EF orientation simulations. The time-dependent EF (44) was implemented as

$$E(t) = E_0 \exp\frac{-(t-t_0)^2}{2\sigma^2} \times H(t_0 - t) + E_0 \times H(t - t_0), \quad (1)$$

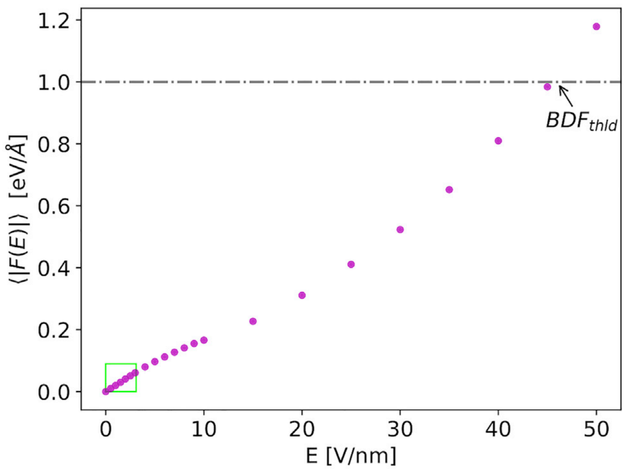

FIGURE 2 Ab initio simulations. Mean value of the force exerted on Trp-cage atoms, defined in Eq. 3, is shown as a function of the electric field strength. The region of interest for the classical MD simulations is presented in the inset.

where $H(t)$ is the Heaviside function, $t_0 \in [0, 2, 5, 9]$ ns and $E_0 \in [0.1, 0.2, 0.5, 0.8, 1.0, 1.5, 2.5, 3.0]$ V/nm. The direction of the EF was set to be parallel to the x axis of the simulation box. For the simulations in which $t_0 \in [0, 2, 5]$ ns, the duration was set to 10 ns and to 14 ns for simulations in which $t_0 = 9$ ns. In total, 320 independent simulations were performed (10 starting structures, four choices of t_0-value, eight EF strengths).

Long-range electrostatic forces in vacuum were captured using no cutoffs for nonbonded interactions. The equations of motion were propagated using the leap-frog integration scheme (45) with a 0.5 fs time step. To reproduce perfect vacuum, neither pressure coupling nor periodic boundary conditions were applied.

RESULTS AND DISCUSSION

Ab initio MD simulations: validation of classical MD approach

Ab initio calculations were performed on the Trp-cage protein. Given the extreme computational effort needed for quantum calculations, we limited the extend of those simulations to comprise only the electronic response, without nuclei dynamics. The main goal of these simulations is to have a quantitative estimation of the order of magnitude of the EF strength needed to break interatomic bonds, as well as validating the use of fixed charges in the classical MD simulations.

In Table 1, some of the most representative equilibrium bond forces within proteins are listed. These values were computed by dividing the tabulated bond energies by the tabulated equilibrium experimental distances. We qualitatively assume that a bond among two atoms is broken when their distance is increased by 25% with respect to their equilibrium distance. We thus define a bond dissociation force (BDF_{thld}) equal to 1 eV/Å as a reasonable lower estimation of the force sufficient to break a covalent bond. This value corresponds to ~80% of the force required to separate two sulfur atoms in a disulfide bond (Table 1).

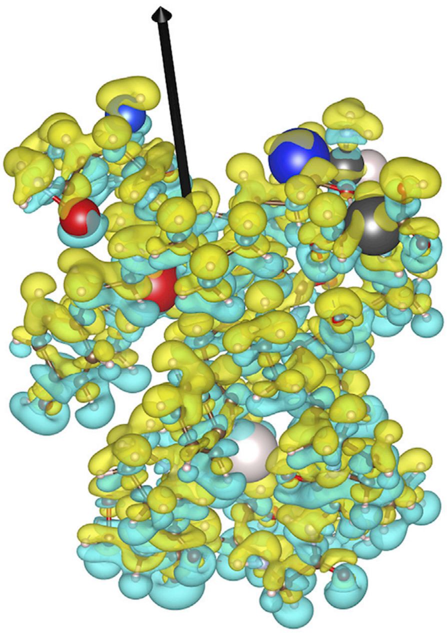

FIGURE 3 Ab initio simulations. The effect of the electric field on the electron distribution in the Trp-cage protein is shown. Displayed is the difference in electron density between a protein unexposed to the field and a protein experiencing a 3 V/nm field. Blue electron density in the figure symbolizes a loss of electron density and green an increase in electron density. Hydrogen atoms are white, oxygen red, nitrogen blue, and carbon black. Atoms that correspond to a maximal decrease of the integrate electron number difference are depicted with atomic radii increased by a factor of 2; atoms that correspond to a maximal increase of the integrate electron number difference are depicted with atomic radii enlarged by a factor of 3. The isosurface level is set to 0.00226 electrons/Å^3. The black arrow denotes the direction of the external electric field.

We assume that the force acting on any atom i in our simulations can be expressed as a sum of three terms, given as

$$F_{i,TOT}(E) = F(d_{CM}) + F(d_i) + F_{i,field}(E). \tag{2}$$

Here, $F(d_{CM})$ is the force depending on the motion of the center of mass of the protein, $F(d_i)$ is the force due to the atomic vibrational state, and $F_{i,field}(E)$ is the contribution to the force given by the interaction with the external EF. For each simulation at the different EF strengths, we calculate the average relative force $\langle|F(E_j)|\rangle$ as

$$\langle|F(E_j)|\rangle = \frac{1}{N}\sum_i^N |F_i(E_j)| - |F_i(E=0)| = \frac{1}{N}\sum_i^N |F_{i,field}(E)|. \tag{3}$$

With this quantity, we isolate the effects induced only by presence of the external EF from the total force at a given E_j. The contributions due to the center-of-mass motion and the particular vibrational state in which the atoms are frozen in are automatically removed. By comparing $\langle|F(E_j)|\rangle$ to BDF_{thld}, it is possible to infer an estimation of the field strength necessary to break a covalent bond. In Fig. 2, we display $\langle|F(E_j)|\rangle$ as function of the simulated EF. The plot shows that the EF strength needed to break atomic bonds is of the order of 45 V/nm. Moreover, one can notice that for all the field strengths used in the classical MD (in the *green inset* of the *graph*), the value of the average force $\langle|F(E_j)|\rangle$ is one order of magnitude lower than BDF_{thld}. This finding proves that the integrity of the protein topology is maintained in that field range and that the accuracy of classical MD is preserved.

TABLE 2 The maximal integrated electron number difference around each site for each atomic species

Atom type	Maximal charge increase	Maximal charge decrease
Hydrogen	0.016	−0.018
Carbon	0.022	−0.013
Nitrogen	0.012	−0.017
Oxygen	0.010	−0.014

Units are in electronic charge e. The integrated difference is defined as "number of electrons on site X at field = 0" − "number of electrons on site X with field = 3 V/nm." The corresponding atoms are highlighted in Fig. 3 by enlarging their radii by a factor of 2 for the maximal decrease and a factor of 3 for the maximal increase.

In Fig. 3, the polarization induced by 3.0 V/nm field on the electron density is shown. At this field strength, the majority of the polarization response is local to the atoms. Hence, not much charge is transferred across the molecule, and the maximal charge increase and decrease in relation to the ground state of a specific atom are relatively small. This validates the approach used in our classical MD simulations, in which the ionic charge is fixed throughout the simulation. In Table 2, we list the maximal increase and decrease in integrated charge on the atoms for which the difference is largest. The corresponding positions of the respective atoms are shown in Fig. 3. The fact that these atoms are not residing at the edge of the protein also indicates the importance of the local polarization above delocalized charge transfer across the molecule, further corroborating the use of classical MD in this context.

Classical MD simulations: orientation in time-dependent EFs

The way the protein responds to the time-dependent EF has been assessed by studying three different observables: degree of orientation, speed of orientation, and the root mean-square deviation (RMSD) of the atomic positions. First of all, to assess the extent of protein orientation we define the degree of orientation as

$$\Theta = 1 - \cos(\theta), \tag{4}$$

where θ is the angle between the EF and the total dipole moment of the molecule. Thus, a fully aligned protein expresses a value of $\Theta = 0$, whereas $\Theta = 1$ corresponds to

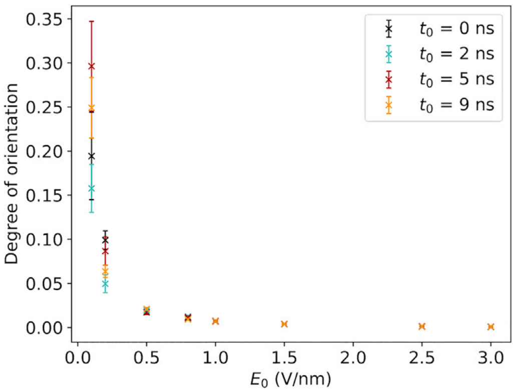

FIGURE 4 Classical simulations. An averaged degree of orientation is shown as a function of the maximal field strength E_0 for different ramp-up times t_0. The different colors refer to the different field implementations (*black*, $t_0 = 0$ ns; *cyan*, $t_0 = 2$ ns; *red*, $t_0 = 5$ ns; and *orange*, $t_0 = 9$ ns).

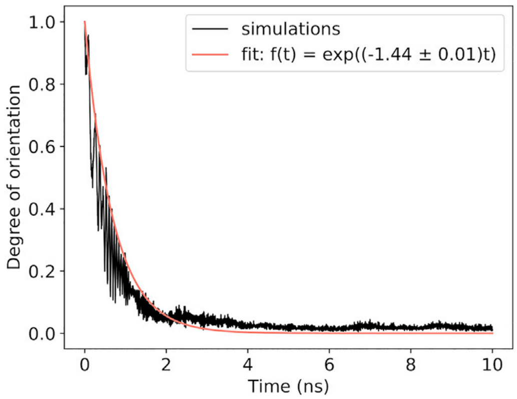

FIGURE 5 Classical simulations. The time evolution of degree of orientation averaged over 10 independent runs for the parameters $E_0 = 0.5$ V/nm, $t_0 = 2$ ns (*black line*) is shown. The orange line is the result of fitting it with the function $f(t) = \exp(-kt)$.

a perpendicular orientation when considering a protein at a particular moment in time. $\Theta = 1$ is, however, also the expectation value for a randomly oriented protein, as parallel and antiparallel orientations then are equally likely and the average $\cos(\theta)$ becomes 0.

In Fig. 4, we show how Θ depends on the EF strength E_0 and the ramping time t_0. Values in Fig. 4 show the average degree of orientation over the last 2 ns of the 10 independent simulations. As one could expect, the stronger the field is, the more the molecule becomes oriented. In Fig. S2, the projection of the dipole moment of the plane perpendicular to the EF vector is depicted. Here, an E_0 of 0.1 V/nm is not enough to orient the protein in the simulation time we explored, and the projection of the dipole moment is equally distributed in the plane, regardless of the value of t_0. For E_0 equal to 0.2 V/nm, although there is not perfect alignment of the EF and the dipole of the molecule, the dipole moment distribution is not completely random. In particular, quite interestingly, the EF implementation with a ramping-up time equal to 2 ns results in a better orientation respect to the other three EF implementations; although not very focused, one can notice a highly populated region corresponding to a spread of $\pm 15°$ with respect to perfect alignment. For field values equal to 0.5 V/nm, the projection of the dipole moment in plane perpendicular to the field is focused in the $\pm 15°$ region, expressing a good alignment between the field vector and the ubiquitin dipole moment. For field strengths greater than or equal to 0.5 V/nm, the differences in the degree of orientation among the ramping times are not resolved within the errors (Figs. 4, S3, and S4). At the end of the simulations, the protein is oriented in a similar way, regardless of the field implementation.

The second observable we monitored in our simulations was the speed of orientation. Although the degree of orientation oscillates significantly, we can observe that there is a clear exponential decay. In Fig. 5, an example of this trend for $E_0 = 0.5$ V/nm, $t_0 = 2$ ns is presented. Here, the black line represents the evolution of $\overline{\Theta}$ over time $(\overline{\Theta}(t) \equiv \langle \Theta(t) \rangle_{replicas})$. The orange line represents a fit on $\overline{\Theta}(t)$, with $f(t) = \exp(-kt)$. We define τ as the time required for the protein to lose 90% of its initial orientation and hence to arrange near parallel to the EF vector:

$$\tau = \frac{\ln(10)}{k}. \tag{5}$$

In Fig. 6, we display the correlation between the orientation time τ for all the simulated field implementations t_0 and E_0. Evidently, a clear dependence between the rate of orientation and the ramping time can be observed: the longer the ramping time is, the more time is needed to orient the structure. Here, τ spans a range of values from 8.3 ns for $E_0 = 0.2$ V/nm and $t_0 = 14$ ns to 3 ps for $E_0 = 3.0$ V/nm and $t_0 = 0$ ns.

The question naturally arises: is there any particular field strength able to orient the protein? To answer this query, we plot, in Fig. 7, $E(\tau)(E_0;t_0)$, namely the value of the EF strength at the time the protein is oriented as a function of the field implementations for the different simulations. Notably, except for the trivial case of the constant field ($t_0 = 0$ ns), the field strength required to align the protein according to this strict orientation criterion seems to be always on the order of 0.5 V/nm, independently of the final field strength of the simulation and the implementations. In other words, an EF strength of 0.5 V/nm is a necessary and sufficient condition to achieve a strong alignment of ubiquitin within 10 ns.

The last important question we assessed regards the structural stability. It is crucial to consider the possible structural changes induced by the presence of an external EF. RMSD computed on Cα atoms gives a measure of how much the structure has changed relative an earlier time point. It is reasonable to define a structure to be preserved if the RMSD value is below 0.5 nm (1), whereas for RMSD values higher than this threshold, we can assume that the protein's initial structure is lost.

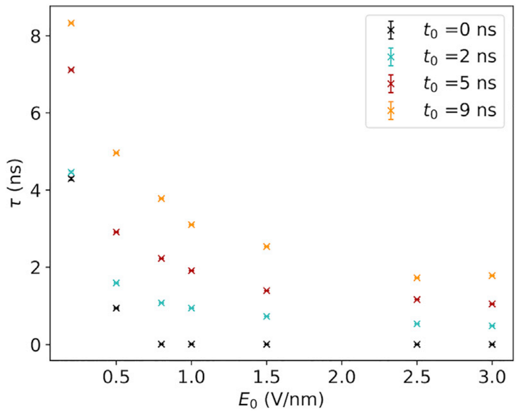

FIGURE 6 Classical simulations. The dependence of time τ (when the protein lost 90% of initial orientation) on E_0 for different ramping time t_0 is shown. The color scheme is the same one described in Fig. 4.

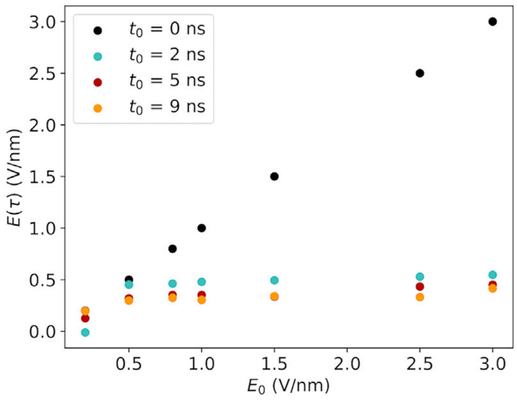

FIGURE 7 Classical simulations. EF strength at the time the protein is oriented is shown as a function of the EF implementation. The color scheme is the same described in Fig. 4.

What is the state of the structures when the proteins become oriented by the field? In Fig. 8, RMSD(τ) (namely the RMSD value at time $t = \tau$) as a function of t_0 and E_0 are displayed for the different field implementations. We can observe that all ramping-up times provide good conservation of the protein structures at the time they are oriented. This assumption is valid for all the values of the EF we tested. We can therefore conclude that in all cases we simulated, the orientation happens before the structure is damaged. An example of this order of events is visualized in Fig. 9.

Lastly, we ask how the protein structures evolve after the EF has reached its maximal strength. To this end, we evaluated the RMSD values of ubiquitin after 5 ns after t_0, that is, we included the trajectory data starting at 5 ns from the beginning of the simulations for the case of $t_0 = 0$ ns, 7 ns for $t_0 = 2$ ns, 10 ns for $t_0 = 5$ ns, and lastly 14 ns for the case of $t_0 = 9$ ns. In SPI, this captures the possibility to acquire the image of the intact protein using the x-ray beam after the molecule traveled in the experimental device. The results of this analysis are presented in Fig. 10, in which for each data point, an average over the 10 replicas was computed. One can observe for EF strengths lower than or equal to 1.5 V/nm, the protein structures are maintained for all the EF implementations. In particular, for an EF lower than or equal to 0.8 V/nm, there are almost no differences among the four different EF implementations. In contrast, EF strengths greater than or equal to 2.5 V/nm yield RMSD values above the 0.5 nm threshold for all the EF implementations, indicating that the structures are no longer native like.

CONCLUSIONS

We have investigated the controlled orientation of proteins in the gas phase using time-dependent external EFs. Time-varying EFs is an inescapable aspect of physical reality of any experimental setup for field orientation, as the proteins must either enter the high-field region from a low-field region or be exposed to a pulsed field that necessarily has a smooth temporal profile at fast timescales, in both cases experiencing an EF that increases over time. As such, this investigation considers an inherent aspect of orientation control of protein using EFs that has been disregarded from earlier studies. Moreover, the pulse profile could, in principle, affect the response of the protein to the applied EF, both in terms of structural damage and orientation, and is an important experimental parameter to consider also for that reason. Our results show that the temporal profile makes little difference for the degree of orientation after 10 ns (Fig. 4). The orientation time τ when the protein is 90% oriented, however, displays a dependence on ramp-up time t_0, which is not unexpected given that higher t_0 implies that the protein will start experiencing a strong field at a later time. More surprisingly, the EF strength at $t = \tau$ ($E(\tau)$) appears to be independent of t_0 and takes a value of ~0.5 V/nm (Fig. 7). We note that this value is comparable with but nonetheless higher than the EF strengths used by Hekstra et al. (3) for inducing motions in protein crystals. We have imposed a rather strict criterion, however, and more relaxed criteria would yield lower values for $E(\tau)$. Orientation of proteins using EFs has been shown to benefit orientation recovery in SPI, but how strong a control is needed for it to be useful is still unknown and an interesting topic for future research. Applications in which only a slight bias is needed will more readily exploit the phenomenon investigated herein, as the lower required field strengths pose less of a challenge to produce. It should be emphasized that using EFs to orient proteins does not provide control over all rotational degrees of freedom. Even for Θ approaching 0, corresponding to perfect alignment with the EF, the protein will be free to rotate around the EF vector. Consequently, EFs cannot be used with applications that require full control over all 3D rotations.

In our investigation, we did not consider residual water molecules on the proteins explicitly. First, the experimental

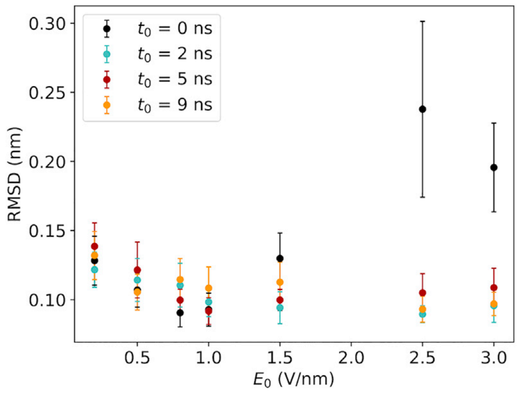

FIGURE 8 Classical simulations. The dependence of RMSD at time $t = \tau$ on E_0 for different ramping time t_0 is shown. The color scheme is the same one described in Fig. 4.

setup might not allow for retained water, but even if it did, their contribution to the net dipole moment would be small. First, only a small number (~10–15) of water molecules are strongly bound to the structure (38). The dipole moment of a single water molecule is furthermore 1.85 D, so even if all water molecules were perfectly aligned, their collective contribution would be small compared to the net dipole of ubiquitin. The water could affect the stability of the protein, but previous investigations have indicated a preserving, and not a destructive, effect (13,14).

We used classical MD to study orientation with EFs. To account for the possibility of bond breaking because of the EF, we carried out quantum-mechanics simulations, which enabled us to identify an approximate limit at 45 V/nm corresponding to the BDF_{thld}, below which bonds remain intact. This value is considerably higher than what has been used in this context before (1,8) and in this study. Importantly, because the interactions that make up protein structures are chiefly the same for all proteins, we can expect our estimate to not be very system dependent. Even without bond breaking, however, the EF could complicate any classical modeling by displacing electrons across the protein, but we found that electrons only shift locally (Fig. 3). Because the electrons remain largely in place and we only considered EF strengths at least an order of magnitude below the bond-breaking limit, we could safely use classical MD for our investigation. The main remaining form of damage that the EFs can cause to the structure of a protein comes from the opposite forces acting on (partial) charges of opposite signs, which can break the noncovalent interactions that keep the fold intact. This happens with very high static EFs, but surprisingly, for nonzero values of t_0, the RMSD of ubiquitin remained low at the moment when the protein had become 90% oriented, meaning that the damage induced by the EF builds up slower than the orientation time of the protein. This feature of "orientation before destruction" can be exploited to probe oriented proteins with unperturbed structures if the time between the EF pulse and the measurement (such as the x-ray pulse in SPI) can be tightly controlled, even for destructive EF strengths.

In our previous work, we noted that longer exposure times also reduced the EF strength needed for orientation, but also for structural loss (1), which entails that the time a protein spends in the field can be expected to modulate the trends we have found here. We also note that we have used a relatively small globular protein as a model system because it is more readily simulated, whereas the systems imaged with SPI so far have been considerably larger. The scattering power scales with the mass of the particle exposed to the beam, and large macromolecular complexes simply give a stronger signal and better signal/noise ratio, making them easier to

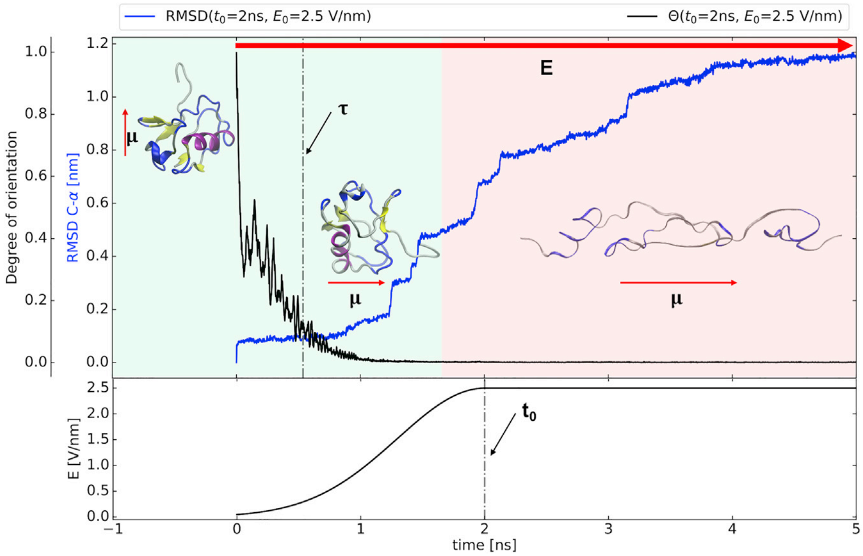

FIGURE 9 Classical simulations. Time evolution of the degree of orientation, RMSD and electric field function for $t_0 = 2$ ns and $E_0 = 2.5$ V/nm simulations is shown. Only the first 5 ns are shown. Each data point shows the result of the averaged values of the 10 replicas. Error bars are not shown for simplicity. The green background represents the part of the simulation in which the ubiquitin structure is preserved (RMSD $\leq$ 0.5 nm). On the contrary, the red background indicates the part of the simulation in which the protein structure is lost. The red arrow denoted with E represents the direction of the external EF; with μ arrows, we represent the direction of the protein dipole. In the insets, cartoon representations of the protein structure at the corresponding time are presented.

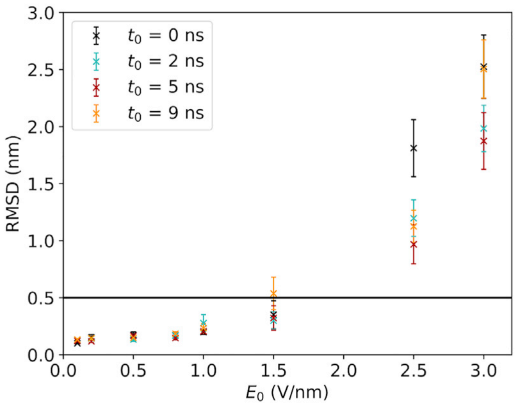

FIGURE 10 Classical simulations. Dependence of RMSD after 5 ns of EF reached its maximal value is shown as a function of E_0 and t_0. The color scheme is the same one described in Fig. 4.

image. Serendipitously, the expected dipole moment of proteins also increases with protein mass (2), making for a stronger interaction with the EF and potentially making large protein complexes orient more readily in the EF. How this combines with the increase in moment of inertia for larger proteins remains to be seen, but in our earlier work, we could discern a trend in which in the lower end of the investigated range of EF strengths, the larger of the proteins we simulated were more oriented. We therefore speculate that for proteins that are amenable for SPI, $E(\tau)$ will take on lower values, especially if longer exposure times are used, matching the EFs that have already been used in other applications (3,31).

SUPPORTING MATERIAL

Supporting material can be found online at https://doi.org/10.1016/j.bpj.2021.07.017.

AUTHOR CONTRIBUTIONS

C.C., E.G.M., and E.D.S. devised the project. C.C., with help from O.G., performed ab initio simulations. H.A., T.M., and A.S. carried out classical simulations. A.S. and E.D.S. analyzed the data. E.D.S., with help from C.C., O.G., E.G.M., and A.S., wrote the article.

ACKNOWLEDGMENTS

We thank Maxim Brodmerkel for comments that greatly improved the manuscript.

This work is a part of the Mass Spectrometry for Single-Particle Imaging of Dipole Oriented protein Complexes (MS SPIDOC) project funded by the European Union's Horizon 2020 Future and Emerging Technologies, FET-OPEN research and innovation program (grant agreement number 801406) for support of ongoing photo activation Ion-Mobility spectrometry–Mass Spectrometry (IM-MS) activities. A.S. acknowledges funding from the Knut and Alice Wallenberg Foundation through the Wallenberg Academy Fellow grant of J. Nilsson. C.C. acknowledges the Swedish Research Council (2018-00740) and the Helmholtz Association through the Center for Free-Electron Laser Science at the Deutsches Elektronen-SYnchrotron (DESY). E.D.S. gratefully acknowledges a postdoctoral fellowship from Carl Trygger Foundation. We acknowledge the use of Uppsala Multidisciplinary Center for Advanced Computational Science provided by the Swedish National Infrastructure for Computing - SNIC (Uppmax project numbers SNIC 2020/15-67, SNIC 2019/8-314, and SNIC 2019/30-47).

REFERENCES

1. Marklund, E. G., T. Ekeberg, …, C. Caleman. 2017. Controlling protein orientation in vacuum using electric fields. *J. Phys. Chem. Lett.* 8:4540–4544.
2. Shvartsburg, A. A., T. Bryskiewicz, …, R. D. Smith. 2006. Field asymmetric waveform ion mobility spectrometry studies of proteins: dipole alignment in ion mobility spectrometry? *J. Phys. Chem. B.* 110:21966–21980.
3. Hekstra, D. R., K. I. White, …, R. Ranganathan. 2016. Electric-field-stimulated protein mechanics. *Nature.* 540:400–405.
4. Hopper, J. T. S., and N. J. Oldham. 2009. Collision induced unfolding of protein ions in the gas phase studied by ion mobility-mass spectrometry: the effect of ligand binding on conformational stability. *J. Am. Soc. Mass Spectrom.* 20:1851–1858.
5. Zhong, Y., L. Han, and B. T. Ruotolo. 2014. Collisional and Coulombic unfolding of gas-phase proteins: high correlation to their domain structures in solution. *Angew. Chem. Int.Engl.* 53:9209–9212.
6. Allison, T. M., E. Reading, …, C. V. Robinson. 2015. Quantifying the stabilizing effects of protein-ligand interactions in the gas phase. *Nat. Commun.* 6:8551.
7. Chandler, S. A., and J. L. Benesch. 2018. Mass spectrometry beyond the native state. *Curr. Opin. Chem. Biol.* 42:130–137.
8. Sinelnikova, A., T. Mandl, …, C. Caleman. 2020. Reproducibility in the unfolding process of protein induced by an external electric field. *Chem. Sci.* 12:2030–2038.
9. Ruotolo, B. T., and C. V. Robinson. 2006. Aspects of native proteins are retained in vacuum. *Curr. Opin. Chem. Biol.* 10:402–408.
10. Breuker, K., and F. W. McLafferty. 2008. Stepwise evolution of protein native structure with electrospray into the gas phase, 10(-12) to 10(2) s. *Proc. Natl. Acad. Sci. USA.* 105:18145–18152.
11. Ruotolo, B. T., K. Giles, …, C. V. Robinson. 2005. Evidence for macromolecular protein rings in the absence of bulk water. *Science.* 310:1658–1661.
12. Koeniger, S. L., S. I. Merenbloom, and D. E. Clemmer. 2006. Evidence for many resolvable structures within conformation types of electrosprayed ubiquitin ions. *J. Phys. Chem. B.* 110:7017–7021.
13. Patriksson, A., E. Marklund, and D. van der Spoel. 2007. Protein structures under electrospray conditions. *Biochemistry.* 46:933–945.
14. Marklund, E. G., D. S. Larsson, …, C. Caleman. 2009. Structural stability of electrosprayed proteins: temperature and hydration effects. *Phys. Chem. Chem. Phys.* 11:8069–8078.
15. Wyttenbach, T., and M. T. Bowers. 2011. Structural stability from solution to the gas phase: native solution structure of ubiquitin survives analysis in a solvent-free ion mobility-mass spectrometry environment. *J. Phys. Chem. B.* 115:12266–12275.
16. Meyer, T., V. Gabelica, …, M. Orozco. 2013. Proteins in the gas phase. *Wiley Interdiscip. Rev. Comput. Mol. Sci.* 3:408–425.
17. Hewitt, D., E. Marklund, …, A. J. Borysik. 2014. A hydrodynamic comparison of solution and gas phase proteins and their complexes. *J. Phys. Chem. B.* 118:8489–8495.
18. Bleiholder, C., and F. C. Liu. 2019. Structure relaxation approximation (SRA) for elucidation of protein structures from ion mobility measurements. *J. Phys. Chem. B.* 123:2756–2769.

19. Bakhtiari, M., and L. Konermann. 2019. Protein ions generated by native electrospray ionization: comparison of gas phase, solution, and crystal structures. *J. Phys. Chem. B.* 123:1784–1796.

20. Aquila, A., A. Barty, …, G. J. Williams. 2015. The linac coherent light source single particle imaging road map. *Struct. Dyn.* 2:041701.

21. Neutze, R., R. Wouts, …, J. Hajdu. 2000. Potential for biomolecular imaging with femtosecond X-ray pulses. *Nature.* 406:752–757.

22. Bogan, M. J., W. H. Benner, …, H. N. Chapman. 2008. Single particle X-ray diffractive imaging. *Nano Lett.* 8:310–316.

23. Spence, J. C. H. 2017. XFELs for structure and dynamics in biology. *IUCrJ.* 4:322–339.

24. Emma, P., R. Akre, …, J. Galayda. 2010. First lasing and operation of an ångstrom-wavelength free-electron laser. *Nat. Photonics.* 4:641–647.

25. Schneidmiller, E. A., and M. V. Yurkov. 2011. Photon beam properties at the European XFEL (December 2010 revision). Technical report. Deutsches Elektronen-Synchrotron (DESY).

26. Seibert, M. M., T. Ekeberg, …, J. Hajdu. 2011. Single mimivirus particles intercepted and imaged with an X-ray laser. *Nature.* 470:78–81.

27. Lundholm, I. V., J. A. Sellberg, …, F. R. N. C. Maia. 2018. Considerations for three-dimensional image reconstruction from experimental data in coherent diffractive imaging. *IUCrJ.* 5:531–541.

28. Kurta, R. P., J. J. Donatelli, …, A. P. Mancuso. 2017. Correlations in scattered X-ray laser pulses reveal nanoscale structural features of viruses. *Phys. Rev. Lett.* 119:158102.

29. Rose, M., S. Bobkov, …, I. A. Vartanyants. 2018. Single-particle imaging without symmetry constraints at an X-ray free-electron laser. *IUCrJ.* 5:727–736.

30. Hantke, M. F., D. Hasse, …, I. Andersson. 2014. High-throughput imaging of heterogeneous cell organelles with an X-ray laser. *Nat. Photonics.* 8:943–949.

31. Wilks, A., M. Hart, …, D. Ruiz-Alonso. 2012. Characterization of a miniature, ultra-high-field, ion mobility spectrometer. *Int. J. Ion Mobil. Spectrom.* 15:199–222.

32. Soler, J. M., E. Artacho, …, D. Sánchez-Portal. 2002. The SIESTA method for ab initio order-N materials simulation. *J. Phys. Cond. Mat.* 14:2745–2779.

33. Neidigh, J. W., R. M. Fesinmeyer, and N. H. Andersen. 2002. Designing a 20-residue protein. *Nat. Struct. Biol.* 9:425–430.

34. MS-SPIDOC. 2021. https://www.ms-spidoc.eu/.

35. Vydrov, O. A., and T. Van Voorhis. 2010. Nonlocal van der Waals density functional: the simpler the better. *J. Chem. Phys.* 133:244103.

36. Hess, B., C. Kutzner, …, E. Lindahl. 2008. GROMACS 4: algorithms for highly efficient, load-balanced, and scalable molecular simulation. *J. Chem. Theory Comput.* 4:435–447.

37. Kaminski, G. A., R. A. Friesner, …, W. L. Jorgensen. 2001. Evaluation and reparametrization of the OPLS-AA force field for proteins via comparison with accurate quantum chemical calculations on peptides. *J. Phys. Chem. B.* 105:6474–6487.

38. Mandl, T., C. Östlin, …, C. Caleman. 2020. Structural heterogeneity in single particle imaging using X-ray lasers. *J. Phys. Chem. Lett.* 11:6077–6083.

39. Vijay-Kumar, S., C. E. Bugg, and W. J. Cook. 1987. Structure of ubiquitin refined at 1.8 A resolution. *J. Mol. Biol.* 194:531–544.

40. Berendsen, H. J. C., J. P. M. Postma, …, J. R. Haak. 1984. Molecular dynamics with coupling to an external bath. *J. Chem. Phys.* 81:3684–3690.

41. Jorgensen, W. L., J. Chandrasekhar, …, M. L. Klein. 1983. Comparison of simple potential functions for simulating liquid water. *J. Chem. Phys.* 79:926–935.

42. Breuker, K., H. Oh, …, F. W. McLafferty. 2002. Detailed unfolding and folding of gaseous ubiquitin ions characterized by electron capture dissociation. *J. Am. Chem. Soc.* 124:6407–6420.

43. Oh, H., K. Breuker, …, F. W. McLafferty. 2002. Secondary and tertiary structures of gaseous protein ions characterized by electron capture dissociation mass spectrometry and photofragment spectroscopy. *Proc. Natl. Acad. Sci. USA.* 99:15863–15868.

44. Caleman, C., and D. van der Spoel. 2008. Picosecond melting of ice by an infrared laser pulse: a simulation study. *Angew. Chem. Int.Engl.* 47:1417–1420.

45. Van Gunsteren, W. F., and H. J. Berendsen. 1988. A leap-frog algorithm for stochastic dynamics. *Mol. Simul.* 1:173–185.

46. Huheey, J. E., E. A. Keiter, …, O. K. Medhi. 2006. Inorganic Chemistry: Principles of Structure and Reactivity. Pearson Education, India.

47. McDaniel, D. H., B. E. Douglas, and J. Alexander. 1983. Concepts and Models of Inorganic Chemistry. John Wiley, New York.

Biophysical Journal, Volume *120*

Supplemental information

Protein orientation in time-dependent electric fields: orientation before destruction

Anna Sinelnikova, Thomas Mandl, Harald Agelii, Oscar Grånäs, Erik G. Marklund, Carl Caleman, and Emiliano De Santis

Manuscript submitted to **Biophysical** *Journal*
Article

Supporting Material: Protein orientation in time-dependent electric fields: Orientation before destruction

A Sinelnikova[1], T Mandl[1,2], H Agelii[1], O Grånäs[1], E G Marklund[3], C Caleman[1,4], E De Santis[1,3,*], Anna Sinelnikova[1], Thomas Mandl[1,2], Harald Agelii[1], Oscar Grånäs[1], Erik G. Marklund[3], Carl Caleman[1,4], and Emiliano De Santis[1,3,*]

[1]Department of Physics and Astronomy, Uppsala University, Box 516, SE-751 20 Uppsala, Sweden
[2]University of Applied Sciences Technikum Wien, Höchstädtplatz 6, A-1200 Wien, Austria
[3]Department of Chemistry – BMC, Uppsala University, Box 576, SE-751 23 Uppsala, Sweden
[4]Center for Free-Electron Laser Science, DESY, Notkestrasse 85, DE-22607 Hamburg, Germany
[*]Correspondence: emiliano.desantis@physics.uu.se

ab initio MD simulations

Table S1: Average relative force, $\langle|F(E)|\rangle$, as a function of the electric field strength. Values are given in eV/Å units.

**Electric field**	$\langle\|F(E)\|\rangle$
0.0	0.000
0.5	0.010
1.0	0.020
1.5	0.030
2.0	0.041
2.5	0.051
3.0	0.061
4.0	0.080
5.0	0.097
6.0	0.112
7.0	0.127
8.0	0.141
9.0	0.155
10.0	0.166
20.0	0.311
25.0	0.411
30.0	0.523
35.0	0.652
40.0	0.810
45.0	0.984
50.0	1.179

Classical MD simulations

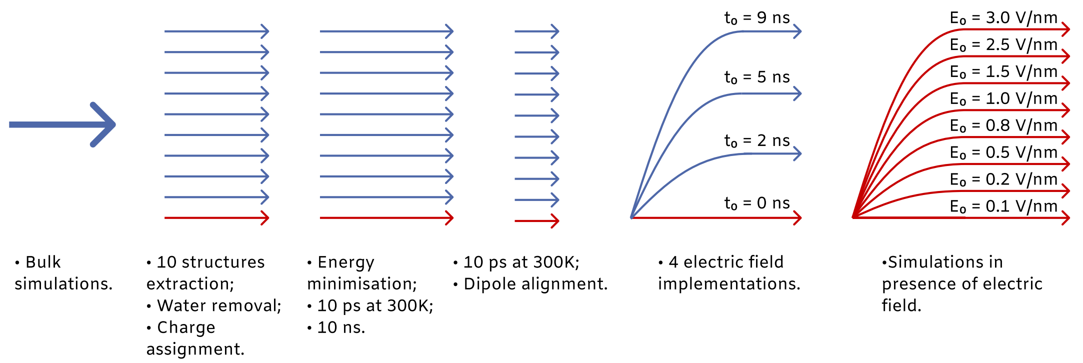

Figure S1: The schematic representation of performed classical MD simulations.

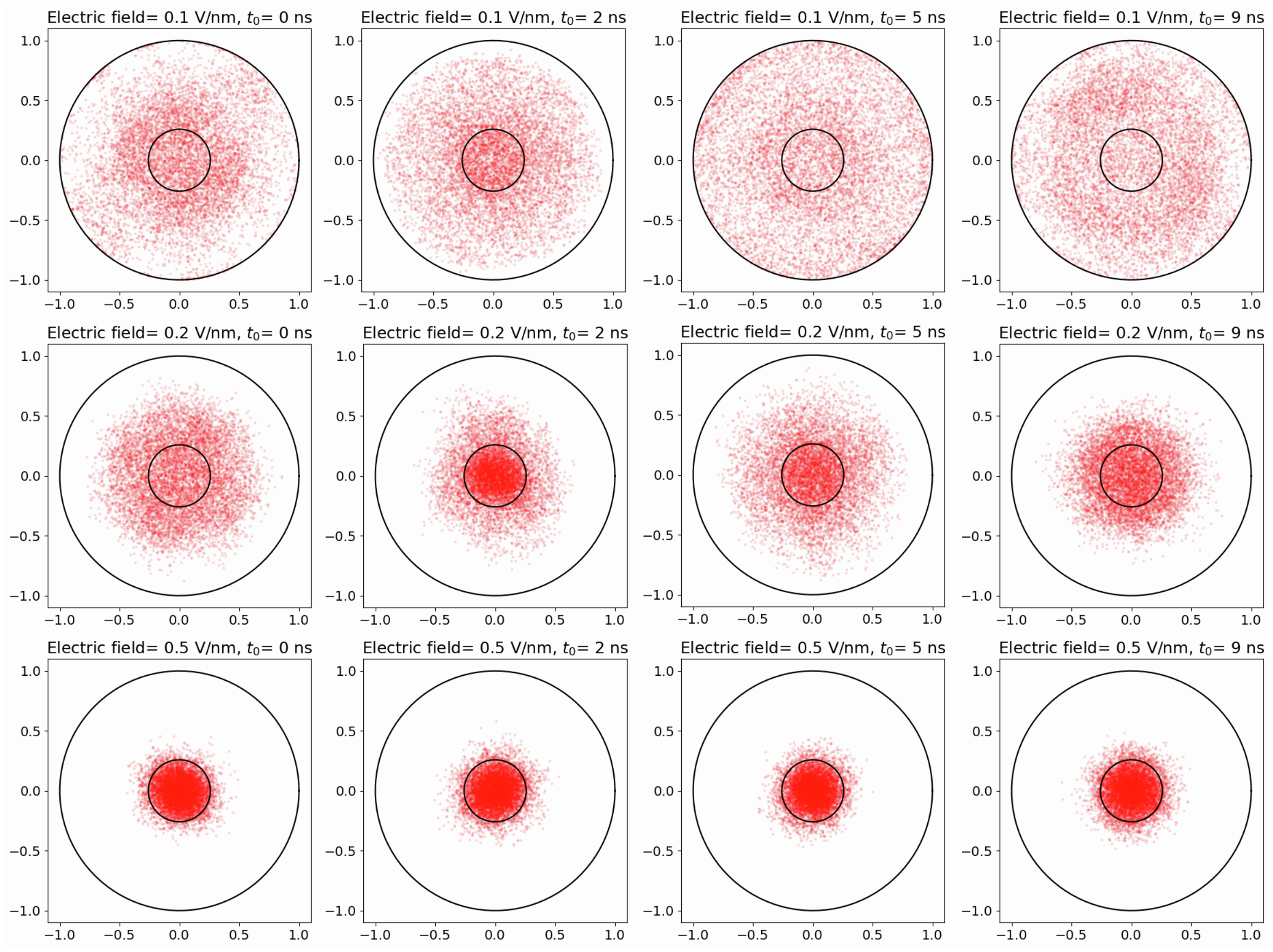

Figure S2: Scatter plot of the projection of the dipole moment on the yz plane for the simulations with electric field strength of 0.1 V/nm (first row), 0.2 V/nm (middle row) and 0.5 V/nm (lower row) . Each point of every panel refers to a frame taken in the last 2 ns of simulations for all the ten replicas. Each panel contains 8000 points (the frames are taken every 2.5 ps of simulations). The circle drawn in the center of the panels represent an uncertainty of ± 15 degrees respect to a perfect alignment.

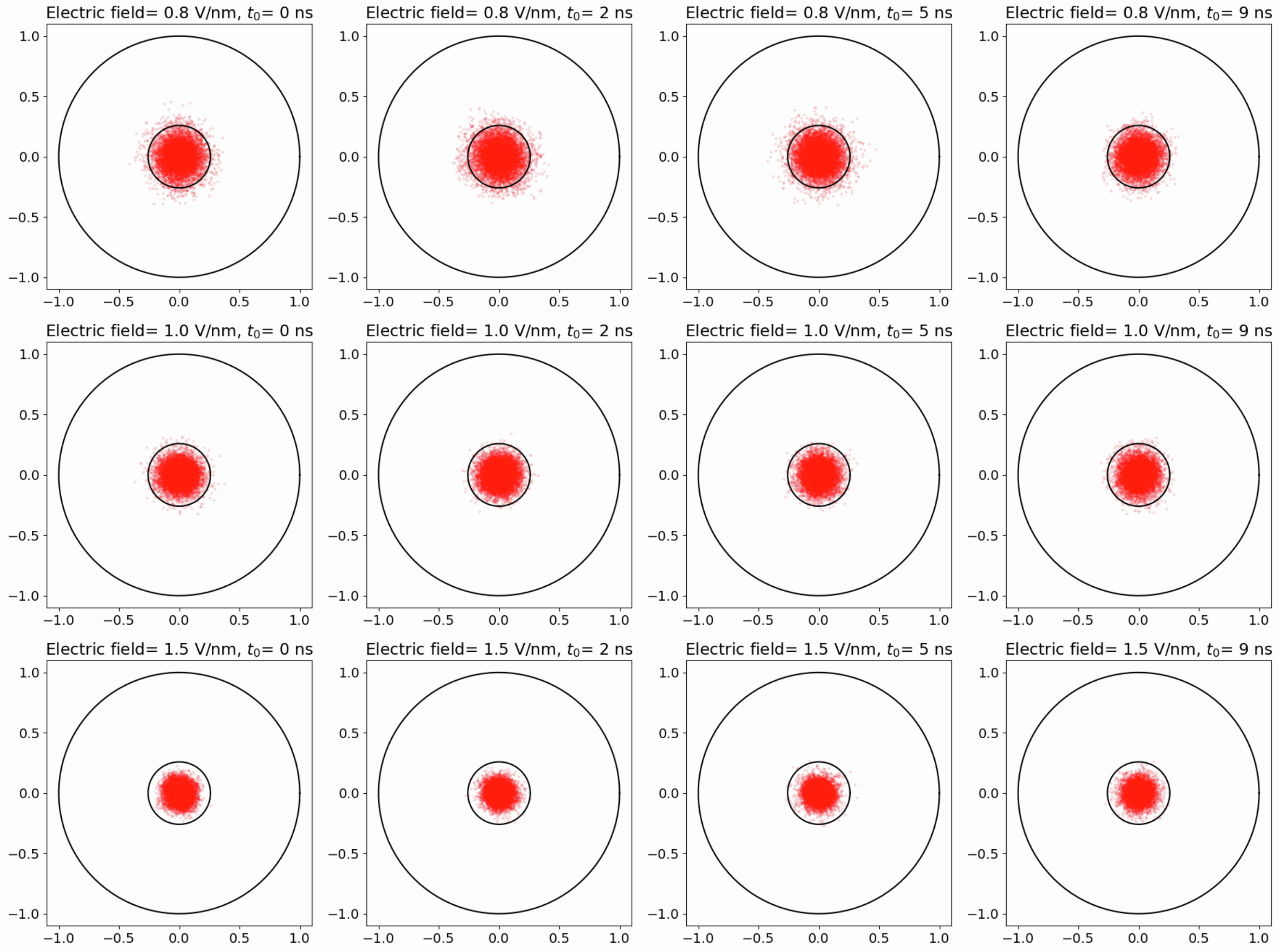

Figure S3: Scatter plot of the projection of the dipole moment on the yz plane for the simulations with electric field strength of 0.8 V/nm (first row), 1.0 V/nm (middle row) and 1.5 V/nm (lower row) . Each point of every panel refers to a frame taken in the last 2 ns of simulations for all the ten replicas. Each panel contains 8000 points (the frames are taken every 2.5 ps of simulations). The circle drawn in the center of the panels represent an uncertainty of ± 15 degrees respect to a perfect alignment.

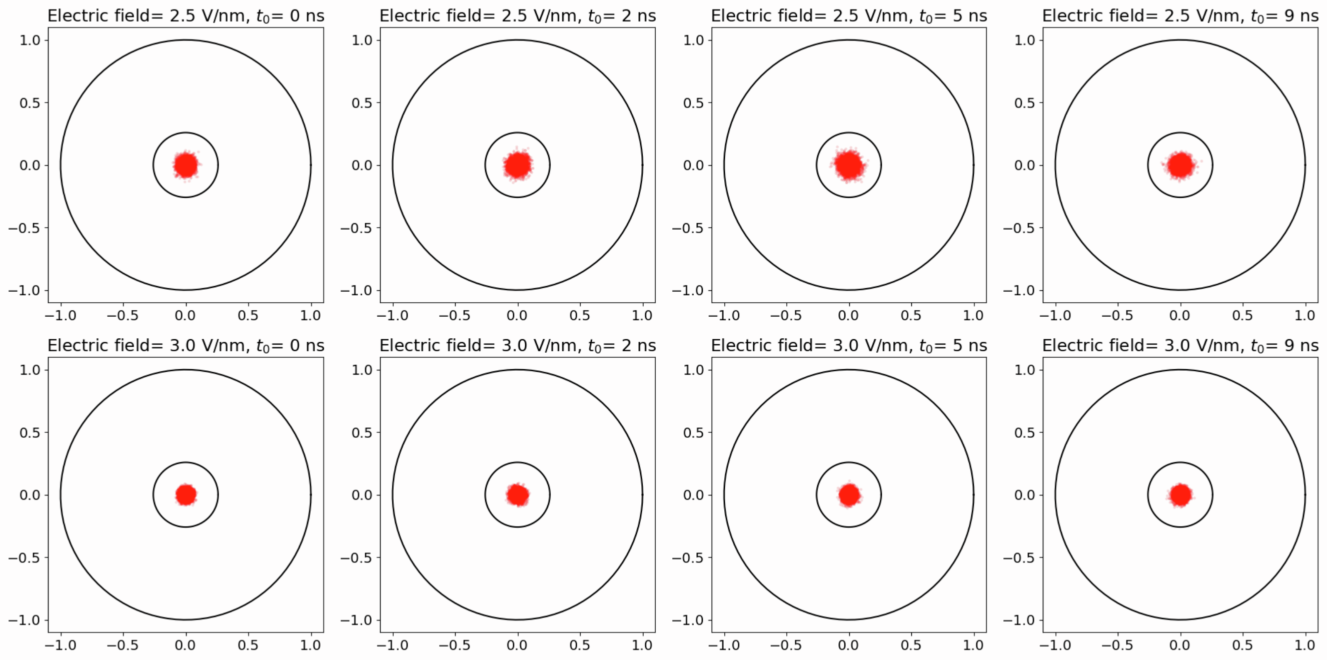

Figure S4: Scatter plot of the projection of the dipole moment on the yz plane for the simulations with electric field strength of 2.5 V/nm (first row) and 3.0 V/nm (lower row) . Each point of every panel refers to a frame taken in the last 2 ns of simulations for all the ten replicas. Each panel contains 8000 points (the frames are taken every 2.5 ps of simulations). The circle drawn in the center of the panels represent an uncertainty of ± 15 degrees respect to a perfect alignment.
